# Understanding the Effect of Structural Diversity in WRKY Transcription Factors on DNA Binding Efficiency through Molecular Dynamics Simulation

**DOI:** 10.3390/biology8040083

**Published:** 2019-11-04

**Authors:** Akshay Singh, Ajay Kumar Sharma, Nagendra Kumar Singh, Humira Sonah, Rupesh Deshmukh, Tilak Raj Sharma

**Affiliations:** 1 National Agri-Food Biotechnology Institute (NABI), Mohali 140306, Punjab, India; akshaybioinfo@gmail.com (A.S.); biohuma@gmail.com (H.S.); rupesh0deshmukh@gmail.com (R.D.); 2 Dr. A.P.J. Abdul Kalam Technical University, Lucknow, Uttar Pradesh 226031, India; 3 Meerut Institute of Engineering and Technology, Meerut, Uttar Pradesh 250005, India; aks.ajayksharma@gmail.com; 4 National Institute for Plant Biotechnology (NIPB), New Delhi 110012, India; nksingh@nrcpb.org

**Keywords:** pigeonpea, DNA binding, molecular modeling, WRKY transcription factor, DBD, molecular dynamics simulation, protein–DNA interaction

## Abstract

A precise understanding of the molecular mechanism involved in stress conditions has great importance for crop improvement. Biomolecules, such as WRKY proteins, which are the largest transcription factor family that is widely distributed in higher plants, plays a significant role in plant defense response against various biotic and abiotic stressors. In the present study, an extensive homology-based three-dimensional model construction and subsequent interaction study of WRKY DNA-binding domain (DBD) in CcWRKY1 (Type I), CcWRKY51 (Type II), and CcWRKY70 (Type III) belonging to pigeonpea, a highly tolerant crop species, was performed. Evaluation of the generated protein models was done to check their reliability and accuracy based on the quantitative and qualitative parameters. The final model was subjected to investigate the comparative binding analysis of different types of WRKY–DBD with DNA-W-box (a cis-acting element) by protein–DNA docking and molecular dynamics (MD) simulation. The DNA binding specificity with WRKY variants was scrutinized through protein–DNA interaction using the HADDOCK server. The stability, as well as conformational changes of protein–DNA complex, was investigated through molecular dynamics (MD) simulations for 100 ns using GROMACS. Additionally, the comparative stability and dynamic behavior of each residue of the WRKY–DBD type were analyzed in terms of root mean square deviation (RMSD), root mean square fluctuation (RMSF)values of the backbone atoms for each frame taking the minimized structure as a reference. The details of DNA binding activity of three different types of WRKY–DBD provided here will be helpful to better understand the regulation of WRKY gene family members in plants.

## 1. Introduction

The WRKY transcription factors represent one of the largest gene families across the plant kingdom and is predominantly involved in the regulation of stress tolerance mechanisms [1]. The first cDNA encoding a WRKY DNA binding peptide (SPF1) was recognized in sweet potato [2]. Since then, WRKY-encoding genes have been identified in various plant species such as rice [3], *Arabidopsis thaliana* [4], poplar [5], cucumber [6], pepper [7], cotton [8], potato [9], soybean [10], chickpea [11]. The WRKY family have been involved in various physiological as well as developmental processes, for instance: seed germination [12], plant growth [13], seed and trichome development [14,15], panicle development [16], secondary metabolism [17], leaf senescence [18], bud and floral differentiation [19], and hormone signaling [20,21]. The WRKY family is also involved in responses to a range of biotic stressors like, drought, salinity [22,23], heat [24], cold [25], and abiotic stressors, such as bacteria [26], nematodes [27], fungi [28], viral pathogen resistance [29].

The name WRKY is due to the presence of a well-conserved WRKY DNA binding domain (DBD) at N-terminal [30]. The WRKY protein also harbors a unique C2H2 zinc finger motif at their C-terminal [30]. The core DNA-binding region in the WRKY protein is a heptapeptide WRKYGQK motif. Different variants of this heptapeptide motif like WRKYGEK, WRKYGKK, WRKYGRK and WKKFEDK in pigeonpea and as WRKYGQE, WRKYGQK, LRKYGPK, WRKDHQK, WRKYGEK, WLKYGKK, GRKYGEK, WMKYGQK,WSKYGQK, WIKYGQK, WKRYGQK, WTKYGQK, WRKYGQN, WRKYGKK, WRICGQK, WKKYGQK, WRMCGQK, and WRKYSEK has been reported in other crop plants [31,32]. The WRKY proteins majorly classified into three groups named as group (I, II, and III), based on the number of WRKY domain, and a zinc finger motif present. The group I proteins have two WRKY domains as well as C2H2 type zinc-finger motifs. Whereas, group II and group III has only one WRKY domain, with a C2H2 type zinc-finger motif in group II, and aC2HC type zinc-finger motif in group III [33,34].

The WRKY domain binds to the cis-regulatory element W-box, (C/T)TGAC(T/C), present at the promoter region of downstream genes, which regulates the signaling cascade [35].The interaction of protein and DNA plays a key role in the function of regulatory proteins, such as the regulation of gene expression, DNA replication, and repair. The phenomenon of binding to specific DNA sequences with a specific protein domain sequence is very complex; the precise mechanism behind the interaction is difficult to predict. Experimental molecular biology approaches have several limitations, for example, how does the interaction take place among the WRKY DNA-binding domain and the target cis-acting element. To overcome these limitations, the use of cost-effective and time-efficient computational approaches such as molecular dynamics (MD) simulations looks promising. To examine the steadiness and dynamics of the predicted DNA–protein docked complexes, numerous computational methods have evolved over the last decade [36]. Docking is required to accelerate the process of information retrieval and to restrict the search criteria for investigational protocols. One of the most efficient methods is comparative modeling of transcription factors which provide detailed information about protein–protein and protein–DNA interactions [37,38]. The crystal structure of WRKY1, WRKY4, and WRKY–DNA (W-box) complex from *A. thaliana* and WRKY45-DNA (the W-box) complex from rice, were determined by using an NMR method [35,39,40,41]. Moreover, protein modeling as well as molecular docking of WRKY3, WRKY4 proteins in tomato [42], WRKY19, WRKY34, WRKY46 of barley [31], AP2/EREBP the protein of barley [43] and NAC1 of *Arabidopsis* [44] have also been studied extensively. The study of WRKY is particularly vital in plant species capable of thriving against adverse conditions like pigeonpea (*Cajanus cajan* L.).

Pigeonpea, a legume food crop, rich in proteins, minerals, and vitamins, is a principle food component of the daily diet for people belonging to semi-arid tropical regions of the word. Pigeonpea belongs to the Fabaceae family and has a diploid (2n = 22) genome of an approximate size of 858 Mbp. Pigeonpea crop has vital importance in food and nutritional security since it provides a rich source of proteins, vitamins, and minerals for more than a billion of the population in the developing world [45,46]. According to Food and Agriculture Organization [47] statistics, the estimated common cropping area under the pigeonpea cultivation was approximately 7.03 million hectares in 2018 with a production of 4.89 million tons and an average yield of 695Kg/ha. Considering the cropping area and the production of pigeonpea, India ranks first in the world, which accounts for approximately sixty-six percent of total pigeonpea production. Most pigeonpea cultivation is under rain-fed conditions, which frequently experiences longer dry spells significantly affecting the yield. Similarly, high heat, extreme water regimes, and biotic stress are the major concerns for pigeonpea cultivation. In this regard, an in-depth understanding of the molecular mechanism involved in stress tolerance is crucial for the development of stress-tolerant pigeonpea cultivars. Similarly, the basic understanding of the biological phenomenon will help to expand the knowledge for the improvement of other crop species.

In our present study, we predicted the homology-based 3D model of the WRKY protein using computational methods. In addition, comparative molecular dynamics simulations of different WRKY variants in pigeonpea to facilitate the molecular mechanisms of WRKY transcription factors and the interaction of these WRKY transcription factors with DNA molecules. The present study provides a basis for the understanding of the structural and functional dynamics of WRKY transcription factors in response to stress in pigeonpea.

## 2. Results

### 2.1. Sequence Analysis and Comparative Phylogeny

In the present study, we selected three WRKY–DBDs representing three different variants that belong to different groups [47]. The CcWRKY1belongedto group I, having two WRKY DBDs, one at N-terminal and another at C-terminal, while only the C-terminal WRKY domain took part in the sequence-specific DNA-binding process, thus the C-terminal domain of CcWRKY1 was considered Type I WRKY-DBD. The Type II (CcWRKY51), member of the group II, had a WRKYGKK motif, (a replacement from amino acid glutamine in group I to lysine; Q14K). Similarly, the Type III (CcWRKY70), member of group III, had a WRKYGEK motif (a replacement from amino acid glutamine in group I to glutamic acid; Q14E) was considered. The nucleotide distribution and A-T-G-C density examination by MATLAB Bioinformatics Toolbox revealed the A-T and G-C rich regions throughout the sequences. Subsequently, the percentage of nucleotide distribution was depicted using a pie chart (Figure S1) and dimmers content as a bar diagram. Evaluation of amino acid composition of the selected CcWRKY protein sequence revealed the frequent occurrence of serine, lysine, threonine, glutamic acid, and valine, as compared to other amino acids (Figure S2).

The physicochemical properties such as molecular weight (mol. wt.) of the selected CcWRKY proteins ranged from 7017.84 to 7528.51 kDa, and the instability index (II) ranged from 17.71 to 39.92 which showed Type I was relatively more stable than other proteins. A protein with an instability index ranging less than 40 usually shows higher stability at in vitro conditions, whereas a predicted instability index above 40 suggests the unstable nature of the protein. At the isoelectric point (pI), proteins were compact and stable. The pI of WRKY-DBDs ranged from 8.75–9.47, showing its basic nature (pI>7.0). For improvement of the thermal constancy of globular proteins, aliphatic index (AI) is an important factor, that indicates the stability for a wide range of temperatures. Range of extinction coefficient values 13,200–14,565 reveals the existence of Cys, Trp, and Tyr amino acid residues. The grand average of hydropathicity (GRAVY) ranges of WRKY-DBDs was very lower (−0.933 to −1.523), which pointed to its higher affinity with water molecules (Table S1). The sub-cellular localization of selected WRKYs using a support vector machine-based prediction tool WoLFPSORT server [48] showed their presence in the nuclear region. The zinc finger signature for Type I, Type II, and Type III WRKY domains were different, Type I contained both a CTD zinc-finger structure C-X4-C-X23-H-X-H and a NTD structure C-X4-C-X22-H-X-H, whereas Type II contained C-X4-C-X23-H-X-H and a Type III had C-X7-C-X23-H-X-C-type zinc finger signature.

The knowledge about the secondary structure of the target proteins is critical to explain the protein folding in 3D structure conformations. The comparative secondary structure analysis amongst the target and template sequences showed very strong homology over the entire sequence (Table S2). The secondary structure analysis also supported the primary sequence level analysis of target and templates. The secondary structure conservation also disclosed the reliability of our proposed model based on target–template alignment predicted by the Modeller software. Secondary structure and the disulfide bridges (S–S) in the target proteins were predicted by using PDBsum and CYS_REC programs (Figure S3).

**Figure 1:**
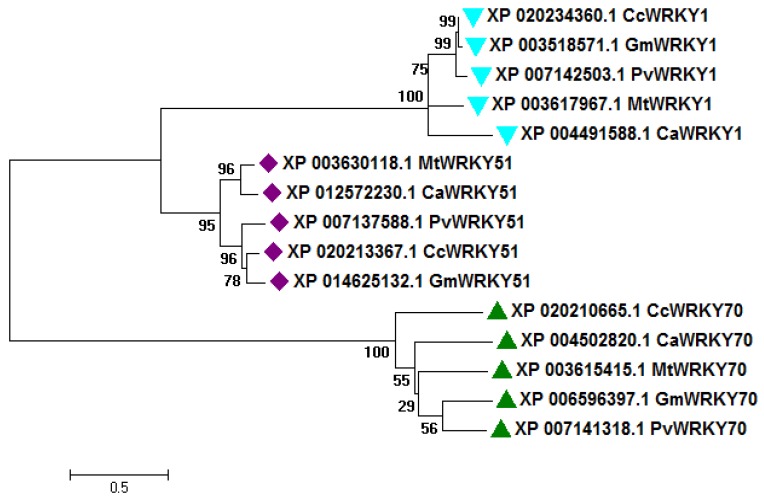
Phylogenetic tree of Type I, II, and III CcWRKY proteins (one representative each) identified in pigeonpea along with homologs from different legume species including *Cajanus cajan* (Cc), *Cicer arietinum* (Ca), *Medicago truncatula* (Mt), *Glycine max* (Gm), and *Phaseolus vulgaris* (Pv). The tree was generated by the maximum likelihood (ML) method with 1000 bootstrap replicates using the MEGA6 software.

The sequence alignment of Type I, Type II, and Type III WRKY DBD within pigeonpea, and with their sequential homologs in *Glycine max*, *Phaseolus vulgaris*, *Medicago truncatula*, and *Cicer arietinum* showing the strong conservation of amino acid residues across the different species (Figure S4). A phylogenetic tree was built using complete peptide sequences of Type I, Type II, and Type III from *C. cajan*, and their sequence homolog from *G. max*, *C. arietinum*, *P. vulgaris*, and *M. truncatula* (Figure 1). The high bootstrap values indicated the conservation of amino acid residues across plant species.

**Figure 2:**
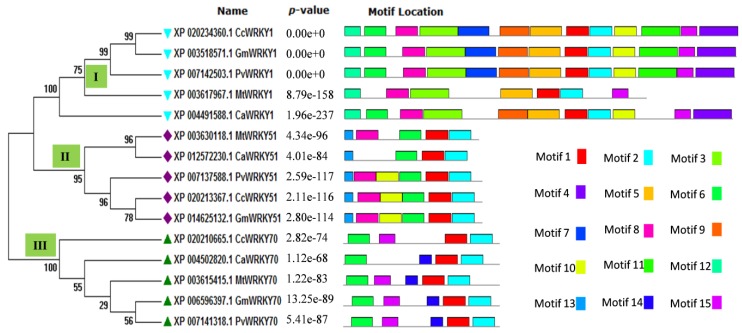
Distribution of conserved motif in respect to the phylogenetic grouping of WRKY transcriptions factor belongs to *Cajanus cajan* (Cc), *Cicer arietinum* (Ca), *Medicago truncatula* (Mt), *Glycine max* (Gm), and *Phaseolus vulgaris* (Pv)**.** The illustration shows the distribution of fifteen predicted conserved motifs in CcWRKY groups. The phylogenetic tree was constructed using the Maximum Likelihood (ML) method with 1000 bootstrap replicates.

For better understanding of the diversity of protein motifs throughout the selected CcWRKYs, the motif distributions were analyzed as a phylogenetic tree for Type I, Type II, and Type III WRKY gene clusters. The statistical significance of the predicted motifs was calculated in terms of their *p*-value. In our results, motif 1 (DGYRWRKYGQKMVKGNTNPRNYYRCSSPGC) represents C-terminal WRKY domain (CTD) which is present in all the fifteen WRKY members, while motif 3 (DGYNWRKYGQKHVKGNEFIRSYYKCTHPNC) represents an N-terminal WRKY domain (NTD) which present only in group I members. Similarly, motif 2 (PVKKHVERASHDSKIVITTYEGQHDHEIPP), which is also a type of WRKY domain, was present in all the members except the CcWRKY50 protein (Figure 2).

Furthermore, the occurrence of additional motifs or un-conserved domains explains their divergence within the group. In group I, four additional protein motifs were present, including motif 4, motif 9, along with motif 12 and motif 14. Motif 5 and motif 7 are specific to group II and III, which leads into the formation of the separate cluster in the phylogenetic tree. Motif 6 and motif eight were found to be present in both groups I and II. Some additional motifs like motif 10, motif 11, motif 13, and motif 19 were present in the entire group I members, while absent in *M. truncatula* (Figure 2).

### 2.2. Construction and Validation of WRKY-DBD Model Variants

The WRKY-DBDs were 60–70 amino acid residues long, extracted from full-length WRKY proteins. The domain characterized by the presence of highly conserved WRKYGQK motif and BLASTP search was considered to retrieve the suitable templates from the Protein Data Bank [49] for modeling the three dimensional (3D) structure of target proteins. The BLASTP results revealed that, three templates (PDB id: 2AYD (AtWRKY1-C-terminal domain), PDB id: 1WJ2 (AtWRKY4-C-terminal domain), and PDB id: 2LEX (AtWRKY4 C-terminal domain and DNA W-box complex) showed highest sequence identity with the targets. Subsequently, the templates were selected to build protein 3D models using Modeller 9.19 program by Ben Webb (University of California, San Francisco, CA, USA). 

**Figure 3:**
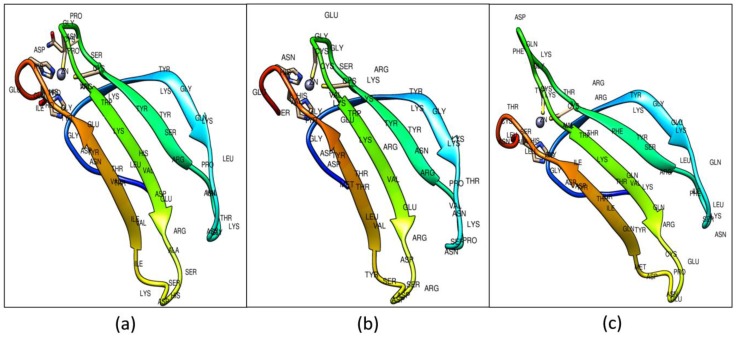
Predicted 3D structure of (**a**) Type I WRKY DBD; (**b**) Type II; and (**c**) Type III WRKY DBDs from pigeonpea. The ligand-binding pockets shown in blue color.

A total of five protein models were selected out of the 30 predicted models for each Type I (CcWRKY1), Type II (CcWRKY51), and Type III (CcWRKY70) proteins. Out of five putative models, the model with the least discrete optimized protein energy (DOPE) score and highest GA341 score was considered as a most thermodynamically stable model and used for further refinement and validation (Figure 3).

To review the conservation across the secondary structure features, the target and template secondary structures were compared with their primary sequences. The modeled WRKY-DBDs consists of four antiparallel β-sheets and a zinc ion. The Type I consists of (β1, Arg-6–Lys-13; β2, Arg-22–Ser-28; β3, Val-34–Ala-41; β4, Ile-47–Glu-53) resides, Type II (β1, Lys-6–Lys-13; β2, Arg-22–Ser-28; β3, Val-34–Asp-41; β4, Tyr-47–Glu-53), and Type III consists (Val-6–Lys-13; β2, Arg-22–Thr-28;β3, Ala-37–Cys-44; β4, Met-50–Ile-56) residues (Figure S5).

**Figure 4:**
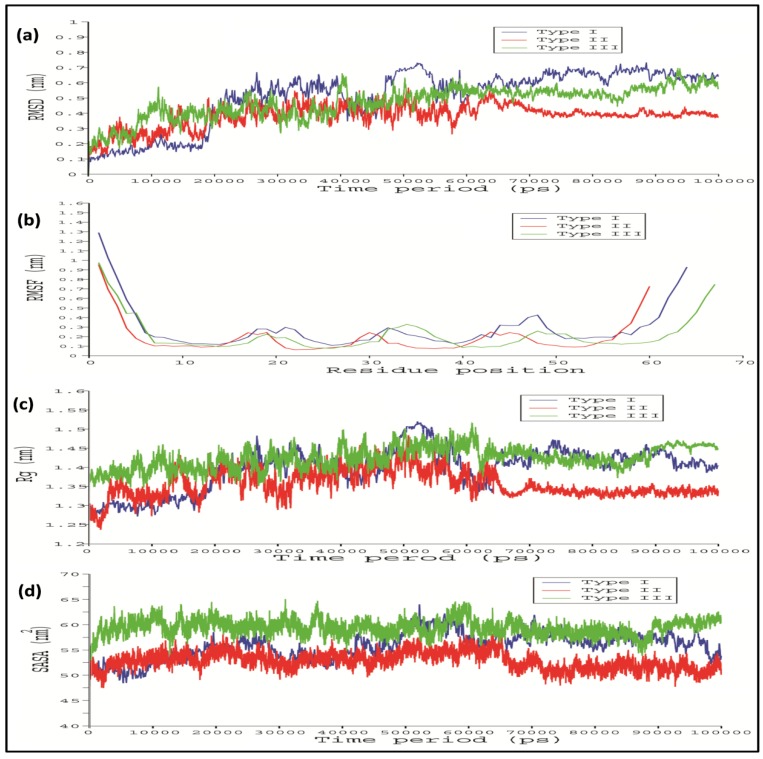
Line diagram showing the (**a**) root mean square deviation (RMSD); (**b**) root mean square fluctuation (RMSF); (**c**) radius of gyration (Rg); and (**d**) solvent accessible surface area (SASA) of Type I, Type II, and Type III CcWRKY DBDs. (Type I in blue, Type II in red, and Type III in green).

To stabilize the stereo chemical properties of the modelled 3D protein model, MD simulation was done for 100 ns using the GROMACS 5.0. From the RMSD analysis, it seems that the Type I WRKY showed a relatively lesser backbone RMSD value 0.1 nm than Type II and Type III which were between 0.1 and 0.2 (Figure 4a). The last 10ns trajectory was extracted from the simulated modes and considered for further docking analysis. Root mean square fluctuation (RMSF) showed the action behavior of each amino acid residue for Type I, Type II, and Type III. The RMSF plot indicated a very similar type of residue fluctuations for Type I, Type II, and Type III, with an average of 1nm to 1.3 nm (Figure 4b). The compactness of protein was measured in terms of the radius of gyration (Rg) score. The Rg plot of protein versus time (300 K) shows the average Rg scores 1.28 nm, 1.3 nm, and 1.38 nm for Type I, Type II, and Type III, respectively (Figure 4c). The Rg score indicated that Type II and Type III show lesser compactness with a high Rg score throughout the simulation process than the Type I WRKY. We also observed the significant differences in the solvent-accessible surface area (SASA) during the simulation run. The SASA calculated in respect to time showed that Type I showed 51.8 nm^2^ while Type II and Type III showed relatively higher SASA values of 52.12 nm^2^and 56.8 nm^2^, respectively (Figure 4d).

Further, the reliability of the protein models was evaluated by PROCHECK server [50]. Ramachandran plots generated through the PROCHECK server gives the full details about the backbone of torsion angels Phi (Φ) and Psi (ψ) dispersal of the amino acids in the protein model. The Ramachandran plot analysis for Type I, Type II, and Type III WRKY-DBDs showed that 98.1%, 98.0%, and 96.7% of the total residues lies in the most favored region. Whereas, 1.9%, 2.0%, and 3.3% were present in the additionally allowed regions, respectively, and no residues were located in generously and disallowed regions (Figure S6).

Further, the model quality was promoted by a high ERRAT score. The overall quality factor for Type I, Type II, and Type III protein models were 75.00, 65.38, and 67.27, respectively, confirming the acceptance of the protein models (Figure S7). The VERIFY3D results showed that greater than 80.0% of the residues averaged a 3D-1D score of ≥0.2, representing the consistency of the predicted models (Figure S8). The qualitative model energy analysis (QMEAN) provides an estimate of the absolute quality of the model by relating it to already-known X-ray crystallography-based solved protein structures (Figure S9). Further, ProSA server [51] (protein structure analysis) was considered for the detection of errors in theoretical and experimental protein models. The z-score showed complete model quality as well as calculated the differences in the total energy of the protein structure with respect to an energy distribution derived from all possible random conformations. The ProSA analysis revealed that the z-scores were very similar for target and templates with a minimum structural error difference, which indicates that the stereo-chemical properties of the protein model coordinates were reliable (Figure S10). Thus, on the basis of qualitative as well as quantitative parameters, the predicted models were found to be appropriate to proceed with further analysis.

The RMSD estimation by the superimposition of the predicted protein model over the already-known experimentally determined structure using the Superpose online server [52] also re-confirms the quality of predicted models. The superimposition of Type I, Type II, and Type III with each other showed that the overall sequence similarity between Type I and Type II: 46/64 (71.9%); Type I and Type III: 33/67 (49.3%), and Type II and Type III: 37/67 (55.2%). A similar type of results was observed when the Type I (CcWRKY1) protein model was superimposed with a template 2AYD (80.3%), the local and global RMSD values were 0.46 Å around backbone and 0.35 Å alpha carbon atoms while the similar template was aligned with Type II (CcWRKY51), the RMSD values for alpha carbons was 0.47 Å and 0.52 Å about backbone atoms and when aligned with Type III (CcWRKY70), the RMSD-values for alpha carbon atoms were1.55 Å and 1.46 Å around for backbone atoms. In contrary, when Type I (CcWRKY1), Type II (CcWRKY51), and Type III (CcWRKY70) protein model were superimposed with the template 1WJ2, we found relatively higher RMSD values for the backbone and alpha carbon atoms (Table S3). The superposition results concluded that the conservancy of WRKY DBD structures along with sequence resemblance across the different WRKY group members. 

### 2.3. Molecular Docking Analysis of WRKY-DBD Variants with DNA-W-box

**Figure 5:**
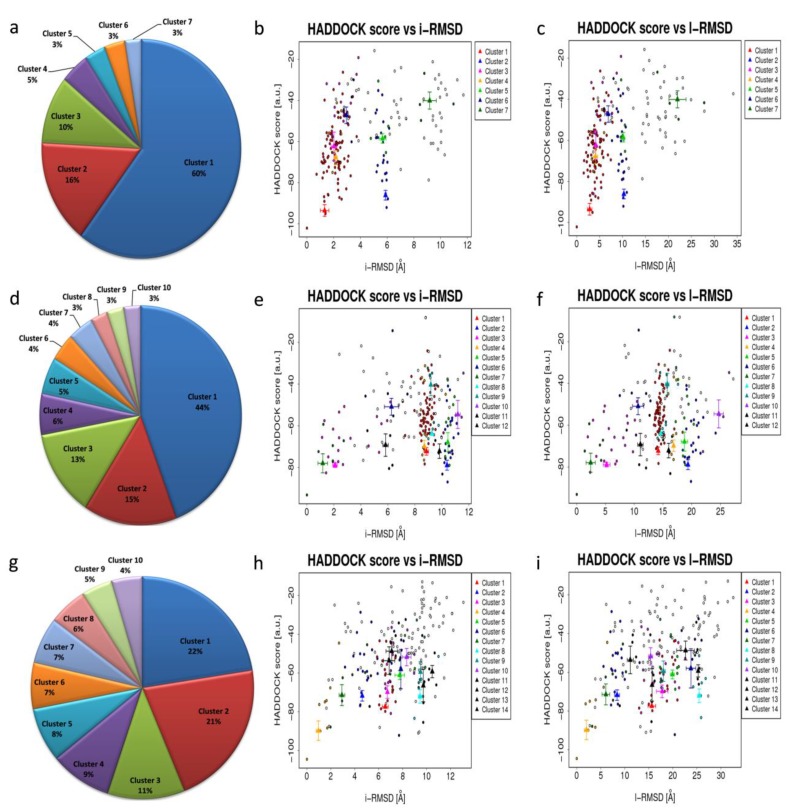
Distribution of HADDOCK clusters estimated for the (**a**) Type I docked complex; (**d**) Type II docked complex and (**g**) Type III docked complex; (**b,c**) HADDOCK scores versus i-RMSD plot and HADDOCK scores versus l-RMSD plot for Type I WRKY–DNA complex; (**e,f**) HADDOCK scores versus i-RMSD plot and HADDOCK scores versus l-RMSD plot for Type II WRKY–DNA complex and (**h,i**) Type III WRKY–DNA complex plot for HADDOCK score versus i-rmsd and l-rmsd.

Protein docking is an essential tool in structural molecular biology, which is used to identify the residues mainly responsible for the interaction among protein–protein or protein–ligand molecules. Docking analysis of WRKY-DBD with the targeted cis-regulatory motifs was studied in several plant species such as rice and *A. thaliana*. The representative protein structure docked with DNA W-box using HADDOCK webserver ver.2.2. server [53] generated seven, twelve, and fourteen clusters for Type I, Type II, and Type III WRKYs, respectively. For Type I, the size of cluster 1 was most significant (60%) and for Type II (44%), whereas the largest cluster in Type III was cluster 1 (22%) (Figure 5). Among all the predicted clusters, the selection of the best cluster was made on the basis of the HADDOCK score. The best scoring complex with lowest HADDOCK score was selected for further analysis.

The HADDOCK score is the sum of van der waals energy, electrostatic energy, desolvation energy as well as restraint violation energies while the z-score shows the dependability of the particular docked complexes from the cluster. For all the docked complexes, HADDOCK score versus i-RMSD and HADDOCK score versus l-RMSD plots were generated. From the predicted clusters of Type I WRKY domain, cluster 1 was considered as the best docked complex having the lowest HADDOCK score of −93.6 +/− 5.6, and z-score of −1.6 (Table S4). For the Type II and Type III, Cluster 2 and Cluster 4 had the lowest HADDOCK scores of −78.9 +/− 4.7 and −89.8 +/− 10.0 and z-scores of−1.2 and −2.3, respectively (Tables S5 and S6).

### 2.4. Comparative Interaction Analysis of Unbound and Bound Complexes Using MD Simulations

To obtain more details and accurate information about the WRKY protein structure as well as WRKY–DNA complex, the WRKY protein–DNA complex was subjected to simulations of 100 nanoseconds (ns). To calculate the stability of Type I, Type II, and III WRKY WRKY–DNA complex, backbone root mean square deviation (RMSD), root mean square fluctuation (RMSF), the radius of gyration (Rg), and solvent accessible surface area (SASA) were examined. Generally, the stability of every WRKY–DNA complex was calculated by analyzing the RMSD profiles for three replications (Supplementary dataset 1).

**Figure 6:**
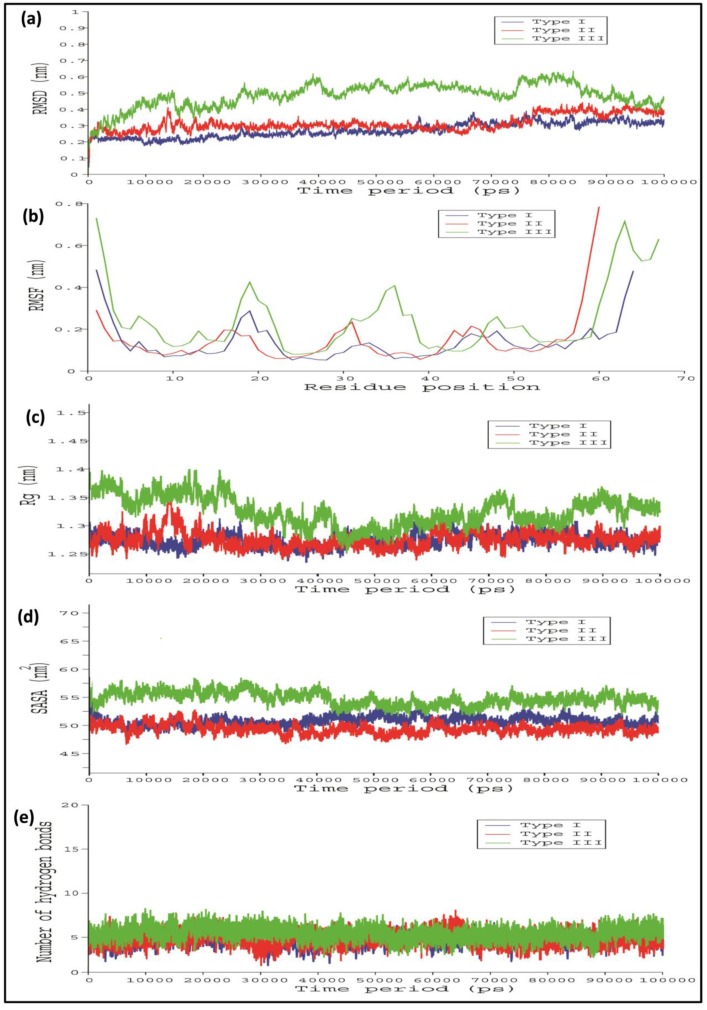
Line graph showing the (**a**) root mean square deviation (RMSD); (**b**) root mean square fluctuation (RMSF); (**c**) radius of gyration (Rg); (**d**) solvent accessible surface area (SASA); and (**e**) number of hydrogen bonds, of Type I, Type II, and III CcWRKY DBD and DNA W-box complexes. (Type I in blue, Type II in red, and Type III in green).

In the case of Type I and Type II WRKY–DNA complexes, it remains stable throughout the entire simulation run, while in Type III, higher variation in RMSD was observed. Furthermore, the Type III WRKY–DNA complex attained overall stability after 85,000 ps (Figure 6a). The RMSF versus time plot showed that heptapeptide amino acid residues of Type III showed slightly higher fluctuation than the Type I and Type II, which enables the stable interaction of Type I WRKY DBD with DNA W-box during whole simulation process as compared to Type II and III (Figure 6b). Subsequently, Type I and Type II complexes were more compact (lower Rg value) than Type III, producing relatively more stable WRKY protein–DNA complexes (Figure 6c). Additionally, RMSF as well as Rg profiles for the Type I and Type II were constant as their RMSD profiles. This significant difference was observed in the solvent accessible surface area (SASA) values during the MD simulation run. The Type I and Type II exhibited SASA value of 50.12 nm^2^ and 51.8 nm^2^, whereas Type III showed relatively higher SASA values of 55.6 nm^2^ (Figure 6d). Significant alterations were noticed in the number of H-bonds in Type I, Type II, and Type III WRKY DBD and DNA during the entire simulation process (Figure 6e). The Type I, Type II, and Type III WRKYs exhibited H-bonds in a range of four to ten throughout the entire simulation process. The average total energy of Type I, Type II, and Type III WRKY WRKY–DNA complexes were −446,044 kJ/mol, −386,285 kJ/ mol, and −394,171 kJ/mol, respectively. The higher energy resulted in greater stability for the Type I WRKY WRKY–DNA complex. The stable Type I, Type II, and Type III complexes were extracted from the last 10 ns for further analysis.

**Figure 7:**
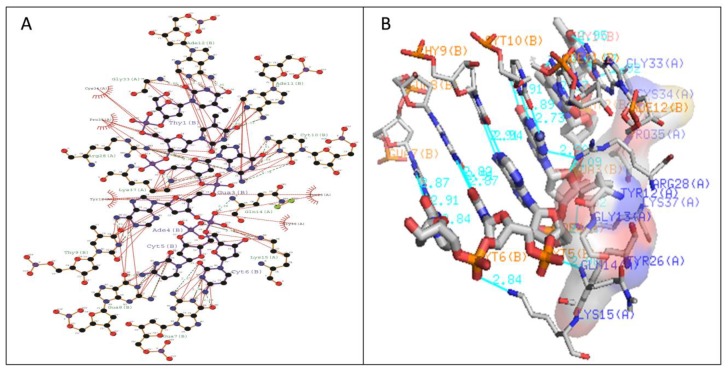
Illustration of (**A**) 2-dimensional and (**B**) 3-dimensional representations of the Type I WRKY protein DNA docked complex. Hydrogen bond interaction of DNA with WRKY protein amino acid residues, Gln14, Arg28, Gly33, and Lys37 (shown by the green line), whereas residues Tyr12, Gly13, Lys15, Tyr26, and Arg28 interacts through hydrophobic bonding (shown in red lines).

**Figure 8:**
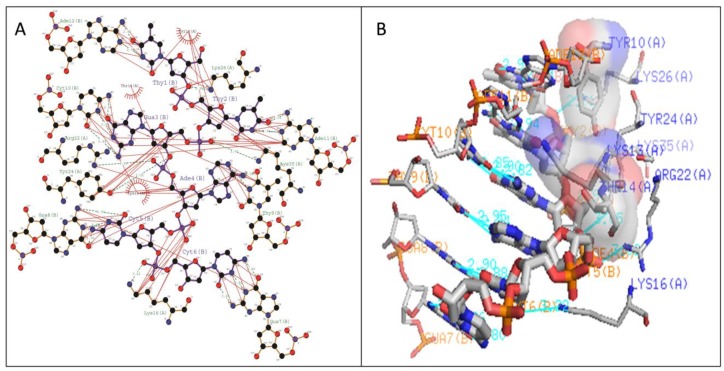
Illustration of (**A**) 2-dimensional and (**B**) three-dimensional representation of the Type II WRKY protein DNA docked complex. Hydrogen bond interaction of DNA with WRKY protein amino acid residues, Arg10, Lys15, and Lys32 (shown by the green line) whereas residues Arg12, Gly14, Lys24, and Arg28 interacts through hydrophobic bonding (shown in red lines).

**Figure 9:**
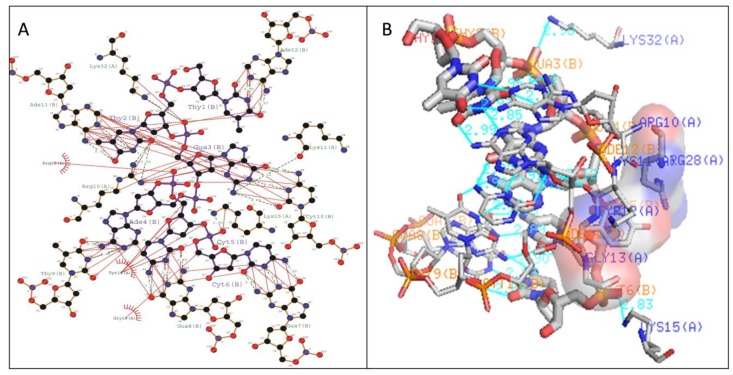
Illustration of (**A**) Two-dimensional and (**B**) 3-dimensional representation of the Type III WRKY protein DNA docked complex. Hydrogen bond interaction of DNA with WRKY protein amino acid residues, Lys16, Arg22, Tyr24 and Lys35 (shown by the green line) whereas residues Lys11, Tyr12, Lys15, Arg33, and Tyr36 interacts through hydrophobic bonding (shown in red lines).

The stabilization of Type I WRKY–DNA complex was due to the establishment of four hydrogen bonds (H-bond) formed with the residues Gln14, Arg28, Gly33, and Lys37 along with three hydrophobic interactions (Tyr12, Gly13, Lys15, Tyr26, and Arg28) (Table 1 and Figure 7). Similarly, the complex of Type II was stabilized by three H-bonds with Arg10, Lys15, and Lys32 residues and four Arg12, Gly14, Lys24, and Arg28 hydrophobic interactions (Table 1 and Figure 8). The complete binding study of the Type III cluster revealed the construction of four H-bonds with Lys16, Arg22, Tyr24, and Lys35 and four hydrophobic interactions Lys11, Tyr12, Lys15, Arg33, and Tyr36 respectively (Table 1 and Figure 9). Generally, the structural feature of the WRKY–DNA complex explains that hydrogen bonds play a significant role to stabilize the protein–DNA complex along with the hydrophobic interactions.

### 2.5. Binding Free Energy Analysis

Comparative binding free energy estimation was carried using the MM-PBSA method to determine the binding affinity of DNA for Type I, Type II, and Type III WRKY DBD. Our results clearly showed that Type I WRKY exhibited relatively lower binding free energy of −443.21 kJ/mol as compared to Type II and Type III with free energy values of −396.86 kJ/mol and −382.12 kJ/mol respectively. Therefore, the Type I had more affinity towards DNA as compared to the Type II and III. It might be due to the loss of contacts between the amino acid residues and nucleic acid.

## 3. Discussion

The WRKY protein plays a diverse role in regulating different physiological processes, such as plant growth, development, plant senescence, signal molecule delivery, biotic, or abiotic stress responses [54]. Several studies on gene expression studies resulting in the revelation of biotic and abiotic stressors in different plant species have confirmed the desired role of WRKY transcription factors in defense response against pathogens [55]. The WRKY transcription factors recognize the W-box(core TTGACC/T motif), which is present at the promoter region of the genes for a preferential binding that regulates the dynamic signaling cascade [56]. The 3D structure and molecular dynamics of WRKY protein and its interaction pattern with DNA W-box in pigeonpea have not been studied yet. The analysis of targeted CcWRKYs at nucleotide as well as protein level describes its nucleotide composition, A-T, C-G loaded region as well as physicochemical properties including mol. weight, distribution of amino acids, theoretical pI, instability index, aliphatic index, and GRAVY index were found in a suitable range for controlling the stability of WRKY protein. The knowledge about the secondary structure of the target proteins is critical to understand the protein folding in 3D-structure conformations. The comparative secondary structure assessment between the target and templates showed strong homology across the entire sequence gives a valid reason for selecting particular templates [57]. The phylogenetic analysis of Type I (WRKY1), Type II (WRKY51), and Type III (WRKY70) of *C. cajan*, and its sequential homolog in *G. max*, *P. vulgaris*, *M. truncatula*, and *C. arietinum* showed strong conservation of core amino acid residues around the WRKY domain disclose their evolutionary significance during their phylogenetic origin. This study revealed the CcWRKYs showing closed evolutionary relatedness with *G. max* followed by *P. vulgaris*, *M. truncatula*, and *C. arietinum*. Conserved motif analysis reveals the presence of a group-specific WRKY domain within the similar groups and the presence of additional motifs or un-conserved domains explains their divergence within the group.

To empathize the molecular mechanisms essentially underlying connections that alter the WRKY transcription factor binding activity, information about the protein sequence and structure is requisite. Comparative protein modeling is considered as one of the most accurate methods for prediction of three-dimensional protein structures, producing appropriate protein models for a broad range of applications in the area of molecular sciences [57]. The greater sequence similarity assures a consistent alignment between the target sequence and the templates. Hence, 3D models for Type I, Type II, and Type III was developed and analyzed through various computational tools to analyze the stereo-chemical quality of our predicted protein model to understand its reliability. Accuracy of the protein model is essential for further analysis like docking, screening, or identification of potential lead compound because the accurateness of the protein model determines the scope of its possible applications. The quality and reliability of the predicted protein models were checked using the Ramachandran plot generated via the PROCHECK server [50]. ThePhi (ɸ) and Psi (ψ) distribution of the amino acids showed 98.1% residues in the most favored region for Type I and 98.0%, 96.7% for Type II, and Type III, suggesting that the predicted protein models fall within the range of known proteins. The VERIFY3D results showed greater than 80.0% of the residues had an average 3D-1D score >0.2, supporting the reliability of the protein models [58], then further ProSA analysis was performed to confirm the quality of models [51], followed by superimposition with experimentally determined structure results. The MD simulation was run for 100 ns to stabilize the Type I, Type II, and Type III WRKY DBD structures.

The docking of protein models with the DNA-W-box using the HADDOCK web server generated seven, twelve, and fourteen clusters for Type I, Type II, and Type III, respectively. The largest cluster size of (60%) was for Type I and for Type II (44%), whereas the largest cluster in Type III is Cluster 1 (22%) (Figure 5). Selection of the best cluster was made on the basis of HADDOCK score, and the complex with the lowest HADDOCK score was selected for further analysis [53]. The cluster 1 of Type I with HADDOCK score of −93.6 +/− 5.6, and Z-score of −1.6, was selected as best docked complex and for the Type II and Type III, cluster 2 and cluster 4 having lowest HADDOCK scores of −78.9 +/− 4.7 and −89.8 +/− 10.0and Z-scores of−1.2 and −2.3 respectively. The stabilization of Type I WRKY–DNA complex is due to the arrangement of four H-bond formed with the Gly13, Gln14, Gly33, and Lys37 residues along with three hydrophobic interactions Gly13, Lys15, and Arg28 (Table 1 and Figure 7). Similarly, the Type II WRKY–DNA complex was stabilized by three H-bonds with Arg10, Lys15, and Lys32 residues and four Arg12, Gly14, Lys24, and Arg28 hydrophobic interactions (Table 1 and Figure 8). The binding study of the Type III cluster revealed the construction of four H-bonds with Lys16, Arg22, Tyr24, and Lys35 and four hydrophobic interactions Lys11, Tyr12, Lys15, Arg33, and Tyr36, respectively (Table 1 and Figure 9). The structural knowledge of the WRKY–DNA complex revealed that hydrogen bonds play a very significant role in stabilizing the WRKY protein–DNA complex as well as hydrophobic interactions.

After the MD simulation of WRKY–DNA complex for 100 ns, the backbone RMSD profiles of Type I, Type II, and Type III were computed to check the stability of the complexes. The Type I and Type II WRKY–DNA complex showing stable throughout the simulation run, while in Type III high variation in RMSD was observed as compared to the Type I and Type II WRKY–DNA complexes, and attains the overall stability after 85,000 ps. The RMSF analysis indicating the higher degree of flexibility of interacting residues for Type II and Type II as compared with the Type I. Subsequently, Type I and Type II WRKY–DNA complexes were more compact (lower Rg value) than Type III, resulted in the stable protein–DNA complex. From the RMSD, RMSF, Rg, and hydrogen bond analysis for WRKY–DNA complexes, it was observed that the Type I complex is more stable than Type II and Type III. The binding energy results indicated that point substitution of glutamine to lysine in Type II and glutamine to glutamic acid substitution in Type III declines the binding affinity of WRKY DBD with DNA, leading to the instability of the complex. Therefore, our results showed that Type I WRKY DBD has more affinity towards DNA as compared to the Type II and Type III variants. It may be due to the loss of interaction between the protein and DNA molecules.

## 4. Materials and Methods

### 4.1. Sequence Retrieval and Analysis

Based on the present analysis, the most commonly occurring variants of WRKY–DBDs in pigeonpea was considered for this study. From the UniProt database [59], the Type I WRKY (WRKY1), Type II, and Type III WRKY-DBD in pigeonpea are named WRKY51, and WRKY70, which are the sequence homologs of AtWRKY1, AtWRKY51, and AtWRKY70, belonging to the WRKY group I, II, and III, and which were selected for this study. The amino acid (Accession no., XP_020234360.1, XP_020213367.1, XP_020210665.1) and nucleotide (Accession no., XM_020378771.1, XM_020357778.1, XM_020355076.1) sequences of the pigeonpea (CcWRKY1, CcWRKY51, and CcWRKY70) were retrieved from the National Centre for Biotechnology Information (NCBI) database [60]. The occurrence of the functional WRKY (DBD) region inCcWRKY1, CcWRKY51, and CcWRKY70 was identified using the NCBI-Conserved Domain Database [61] and the ExPASy-ScanProsite tool [62]. The MATLAB Bioinformatics toolbox [63] was used for sequence analysis to determine the density of nucleotides, AT-GC density, dimercount, base count, and peptide proportion.

### 4.2. Primary and Secondary Structure Analysis

The primary structural properties of the selected peptides were studied using the Expasy-ProtParam tool [64]. Physicochemical properties such as mol. weight, atomic composition, isoelectric point (pI), instability index (II), aliphatic index (AI), and Ggrand average of hydropathicity (GRAVY) were measured. Secondary structure prediction of CcWRKY1, CcWRKY51, and CcWRKY70 was made using self-optimized prediction from multiple alignments (SOPMA) tool [65]. The SOPMA is based on self-optimized prediction approach which has been described to increase the success rate in the secondary structure prediction of target proteins and visualization of the secondary structure by using the PDBsum web server [66]. The CYS_REC program of Soft Berry was used to calculate disulfide bridges (S–S) present in the peptides [67].

### 4.3. Sequence Alignment, Phylogeny, and Potential Motif Analysis

Sequence alignment of Type I, Type II, and Type III WRKY DBDs (CcWRKY1, CcWRKY51, and CcWRKY70), along with their sequential homologs in *G. max*, *P. vulgaris*, *M. truncatula*, and *C. arietinum* were performed. A phylogenetic tree was constructed using the ML method, which is based on the JTT+G+I substitution model having lowest a BIC scores (Bayesian information criterion) with 1000 replicates of bootstrap by MEGA ver.6.0 [68]. The distribution of potential motifs in selected WRKYs were investigated using multiple expectation maximization for motif elicitation (MEME) Suite 5.0.3. [69]. The MEME performs local, gapless, multiple sequence alignment, and predicts significant motifs.

### 4.4. Generation of Protein 3D Models

The selected CcWRKYs with >50% sequence identity with the templates in the BLASTP [70] analysis against RCSB Protein Data Bank was employed for comparative 3D protein structure modeling. Templates PDB ID: 2AYD (C-terminal WRKY domain of AtWRKY1), 1WJ2 (C-terminal WRKY domain of AtWRKY4), and 2LEX (Complex of the C-terminal WRKY domain of AtWRKY4 and W-box DNA), showing the higher sequence identity, query coverage as well as less E-value with the targets. The comparative protein modeling starts with a sequence alignment of a target sequence, and the template whose structure has been already known, the three-dimensional structure of target protein was generated by Modeller 9.19 program [57].

### 4.5. Evaluation of Structural Models

The stability and reliability of the generated protein models were verified using SAVES server version 4 [50]. The server has several assembled programs such as PROCHECK, which investigate the residues in available zones of Ramachandran plots, used to assess the stereochemical quality of the protein model. The ERRAT gives the overall quality factor of the protein, used to check the statistics of the non-bonded interactions among different atoms [71]. Similarly, VARIFY_3D was to determine the compatibility of the atomic model with its own amino acid sequence [58]. Energy minimization of the predicted WRKY proteins was done by using Swiss PDB Viewer 4.1.0. The ProSA Web Server was used in the refinement and validation of the minimized protein structure by measuring the deviation of the total energy of the structure with respect to an energy distribution derived from random conformations [51]. In addition, to determine the structural topology, the modeled C-terminal domain of each Type I, Type II, and Type III WRKY protein models were made to superimpose over the template using SuperPose version 1.0 [52].

### 4.6. Binding Site Prediction

The functionality of a protein sequence was determined by the presence of a well-conserved group of amino acids recognized as a binding site. The binding sites of Type I, Type II, and Type III CcWRKYs were predicted by the COACH server [72], which is a meta-server approach based protein–ligand binding site prediction server. It investigates the ligand binding sites in a target protein by comparing with corresponding binding-specific substructure and sequence profile. The model with the lowest DOPE score and highest GA341 score were chosen for further analysis.

### 4.7. Protein–DNAInteraction

To investigate the molecular connections between WRKY-DBD and DNA W-box, DNA (W-box) was docked to the specific binding sites of WRKY DBDs using HADDOCK web server version 2.2 [53]. The HADDOCK is known for high ambiguity-driven protein–protein docking that calculates docking interfaces for protein–nucleic acid complexes based on experimental knowledge in the form of ambiguous interaction restraints. Ambiguous interaction restraints (AIR) were generated on the basis of active and passive residues in WRKY–DBD. The possible binding sites predicted from the COACH server was designated as active amino acid residues while passive amino acid residues were automatically defined by the program. Ambiguous interaction restraints (AIR) were generated on the basis of lists of selected active and passive residues for both protein and DNA. Further, the illustration and visualization of the docked complexes were analyzed by UCSF Chimera ver.1.11.2 [73] for their interaction studies. The W-box DNA-protein complex was inspected using LigPlot^+^ v.2.1 [74] and PyMol ver.3.2 [75].

### 4.8. Molecular Dynamics (MD) Simulations Analysis

The MD simulation of modeled WRKY–DBD variants and its complexes with DNA-W-box were carried out by using the GROMACS 5.0 software package [76]. For the simulation of bound and unbound WRKY–DBDs AMBER99SB-ILDN proteins, the nucleic AMBER94 force field was applied [77]. A force field is basically like a mathematical equation used to calculate, simulate the binding energy of the atoms. The acronym AMBER stands for assisted model building and energy refinement. For the stability analysis, protein and protein–DNA complexes were solvated into a cubic water box, with a temperature of 300 K which was retain computationally. The systems were solvated using the simple point charge (SPC) water model [78] and sodium and chloride ions were added for neutralization of the entire system. At first, the protein models were simulated to investigate its dynamic behavior and stability without the DNA molecule. The protein–DNA complex was generated using the HADDOCK server. Energy minimization was initially performed when the maximum force was below 1000 kJ/mol/nm, using the steepest descent method for 50,000 cycles, to release steric clashes or conflicting contacts. After the energy minimization step, all systems were employed for a 10,000 ps position restraint dynamics simulation (equilibration phase) under NVT-fixed volume and temperature and NPT-fixed pressure and temperature phase conditions. Finally, the well-equilibrated system was subjected to an MD production run for 100 nanoseconds at 300 K and 1 bar pressure. The stability and dynamic behavior of each residue of the WRKY–DBD variants were also analyzed by plotting backbone root mean square deviation (RMSD) versus time graph. Similarly, the radius of gyration (Rg), root mean square fluctuation (RMSF), solvent accessible surface area (SASA) and principal component analysis were analyzed using GROMACS’ inbuilt utilities g_rmsf, g_gyrate, g_sasa, and g_cover, respectively.

## 5. Conclusions

The 3D structure of the WRKY domain in pigeonpea was predicted through a comparative modeling approach using already-known crystal structure as templates. On the basis of MD simulation, analysis of Type I and Type III showed higher deviation and fluctuations as compared to Type II. From the 100 ns MD simulation of Type I, Type II and Type III WRKY–DNA complexes, it was concluded that the H-bonds and hydrophobic interactions played a significant role in stabilizing the protein–DNA complex. The substitution of amino acid in the conserved domain may alter the interaction behavior of protein DBD with the DNA, which leads to the changed protein function. We conclude that the analysis of WRKY transcription factors and target cis-regulatory elements will be helpful to understand better the effect of different WRKY variants and overall molecular mechanisms regulated by the WRKY family in plants.

## Figures and Tables

**Table 1:** Residues that participated in hydrogen bond and hydrophobic interactions in the WRKY–DNA complexes.

WRKY Variants	Pre-Simulated WRKY–DNA Docked Complex		Post-Simulated WRKY–DNA Docked Complex	
	Hydrogen Bonds	Hydrophobic Interactions	Hydrogen Bonds	Hydrophobic Interactions
Type I	Gln14-DC5 (2.98 Å) Arg28-DG3 (2.9 Å) Arg28-DG3 (3.09 Å) Gly33-DT1 (2.92 Å) Lys37-DG3 (3.02 Å) Lys37-DA4 (2.94 Å)	Tyr12, Lys15, Tyr26, Gly33, Cys34, Pro35, Lys37	Gln14-DC5 (2.98 Å) Arg28-DG3 (2.9 Å) Gly33-DT1 (2.92 Å) Lys37-DG3 (3.02 Å) Lys37-DA4 (2.94 Å)	Tyr12,Gly13, Lys15, Tyr26,Arg28
Type II	Gly11-DA4 (2.87 Å) Lys13-DC6 (2.9 Å) Lys15-DG7 (2.89 Å) Arg22-DT2 (2.8 Å)	Arg10,Lys12,Lys15,Arg22, Tyr25	Arg10-DT2 (2.9 Å) Lys15-DC6 (2.83 Å) Lys32-DG3 (2.98 Å)	Arg12,Gly14,Lys24,Arg28
Type III	Lys11-DC6 (2.87 Å) Tyr12-DA4 (2.8 Å) Lys15-DC6 (2.83 Å) Lys32-DG3 (2.96 Å) Gln35-DC6 (2.9 Å)	Arg10,Lys11, Gly13, Lys32,Arg45,Tyr53,Gln54	Lys16-DC6 (2.9 Å) Arg22-DA4 (3.02 Å) Tyr24-DG3 (2.89 Å) Lys35-DG3 (2.91 Å)	Lys11, Tyr12, Lys15, Arg33,Tyr36

## Supplementary Materials

The following are available online at https://www.mdpi.com/2079-7737/8/4/83/s1, **Figure S1.** Nucleotide distribution pattern of different types of WRKY transcription factors encoding genes. (**A**) Type I WRKY DBD; (**B**) Type II WRKY DBD; (**C**) Type III WRKY DBD, shows the distribution of nucleotides; (**D**) Type I WRKY DBD; (**E**) Type II WRKY DBD; (**F**) Type III WRKYDBD, shows the percentage nucleotide distribution in Type I, II and III WRKYs. **Figure S2.** Frequency distribution of different combinations of nucleotide dimmers observed in genes encoding. (**A**) Type I WRKY DBD; (**B**) Type II WRKY DBD; (**C**) Type III WRKY DBD, and the bar graph shows distribution of different amino acids in DNA binding domain (DBD) of WRKY transcription factor proteins including; (**D**) Type I WRKY; (**E**) Type II WRKT; (**F**) Type III WRKY. **Figure S3.** Topology diagrams showing the secondary structure of Type I, Type II, and Type III WRKY generated by PDBsum web server. Four beta strands are labeled by ‘β’ and represented as arrows. **Figure S4.** Multiple sequence alignment showing strong conservation of amino acid residues across the different species. **Figure S5.** Distribution of β-sheets in (**a**) Type I; (**b**) Type II, and (c) Type III WRKYs of pigeonpea. The C and N represent the C-terminal and N-terminals of the WRKY domain. **Figure S6.** Ramachandran plot of (**a**) Type I WRKY; (**b**) Type II and (**c**) Type III CcWRKY proteins. The most favored region indicated by red color, additionally allowed, generously allowed, and disallowed regions are indicated as yellow, light yellow, and white colors, respectively. **Figure S7.** ERRAT overall quality score analysis of the protein models for Type I, Type II, and Type III WRKY DBDs. **Figure S8.** Varify3D amino acid level quality score of Type I, Type II, and Type III WRKY DBD protein models. **Figure S9.** Qualitative Model Energy Analysis (QMEAN) measurements for protein model quality of Type I, Type II, and Type III WRKY DBDs. **Figure S10.** Comparative qualitative evaluation of Type I, Type II and Type III CcWRKYs performed using ProSA web server, the plot showing the structural error at each amino acid residues in the protein and estimates the overall quality score. The ProSA score for Type I, Type II and Type III were found close to the templates (2AYB and 1WJ2). **Table S1.** Physicochemical characteristics of selected accessions of Type I, Type II, and III WRKYs from pigeonpea. **Table S2.** Comparative secondary structure analysis of target and templates predicted from Self-Optimized Prediction Method with Alignment (SOPMA) Server. **Table S3.** Super imposition of Type-I, Type-II and Type-III WRKY members and also with the templates to calculate the structural closeness in terms of global as well as local RMSD-values that showed the structural conservation of WRKY DBDs. **Table S4.** WRKY–DNA complexes predicted for Type I (CcWRKY1) WRKY-DBD using HADDOCK Server. **Table S5.** WRKY–DNA complexes predicted for Type II (CcWRKY51) WRKY-DBD using HADDOCK Server. **Table S6.** WRKY–DNA complexes predicted for Type III (CcWRKY70) WRKY-DBD using HADDOCK Server. **Supplementary dataset 1.** Comparative analysis of the RMSD profiles of WRKY–DNA complexes for three replications.
