# Supplementary material for: Understanding the Effect of Structural Diversity in WRKY Transcription Factors on DNA Binding Efficiency through Molecular Dynamics Simulation
Source: Biology (Basel). 2019 Nov 4;8(4):83. doi: 10.3390/biology8040083 (PMC6956055; doi:10.3390/biology8040083)

**Figure S11.** Comparative analysis of the RMSD profiles for WRKY-DNA complexes by considering two more replications.
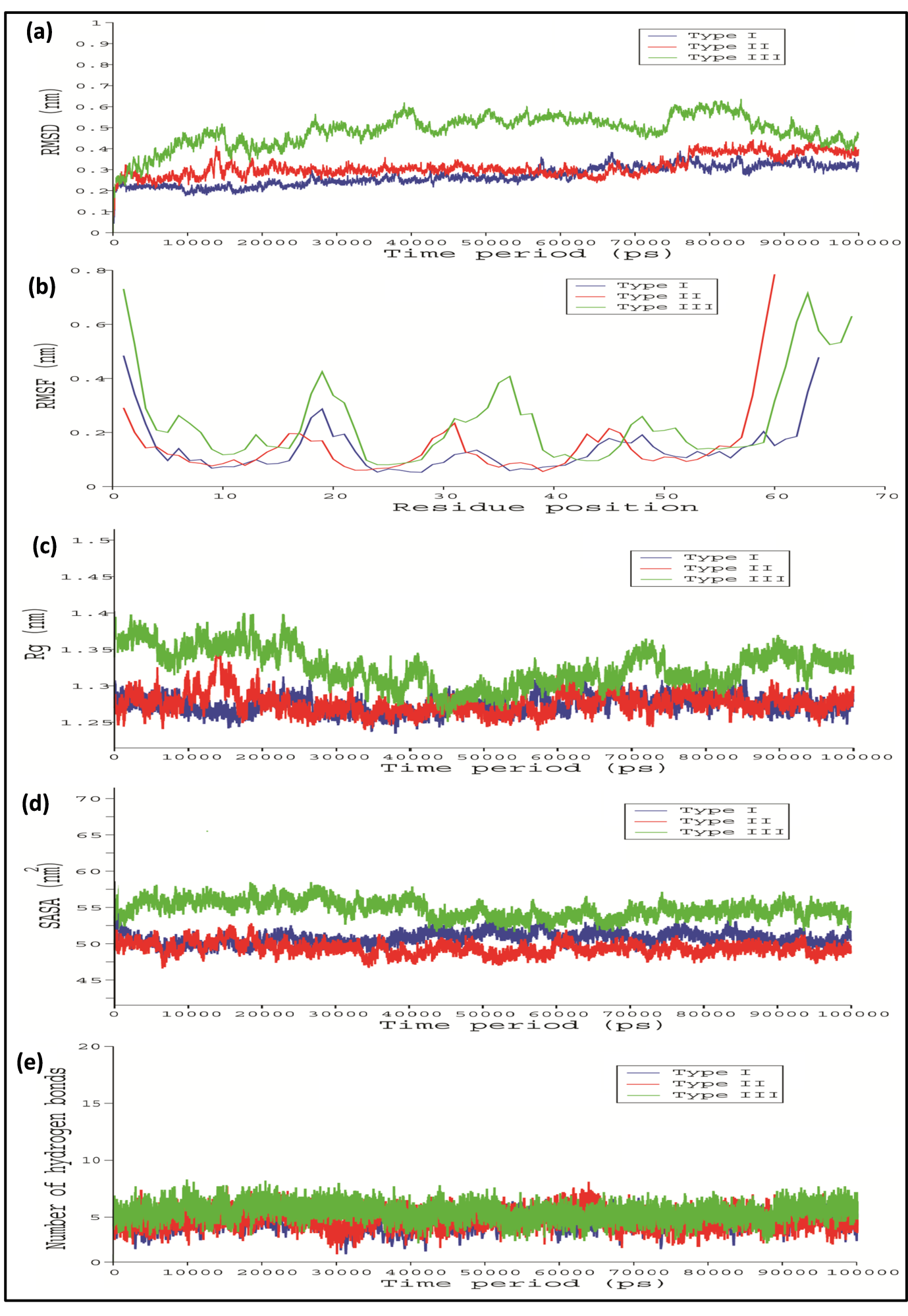


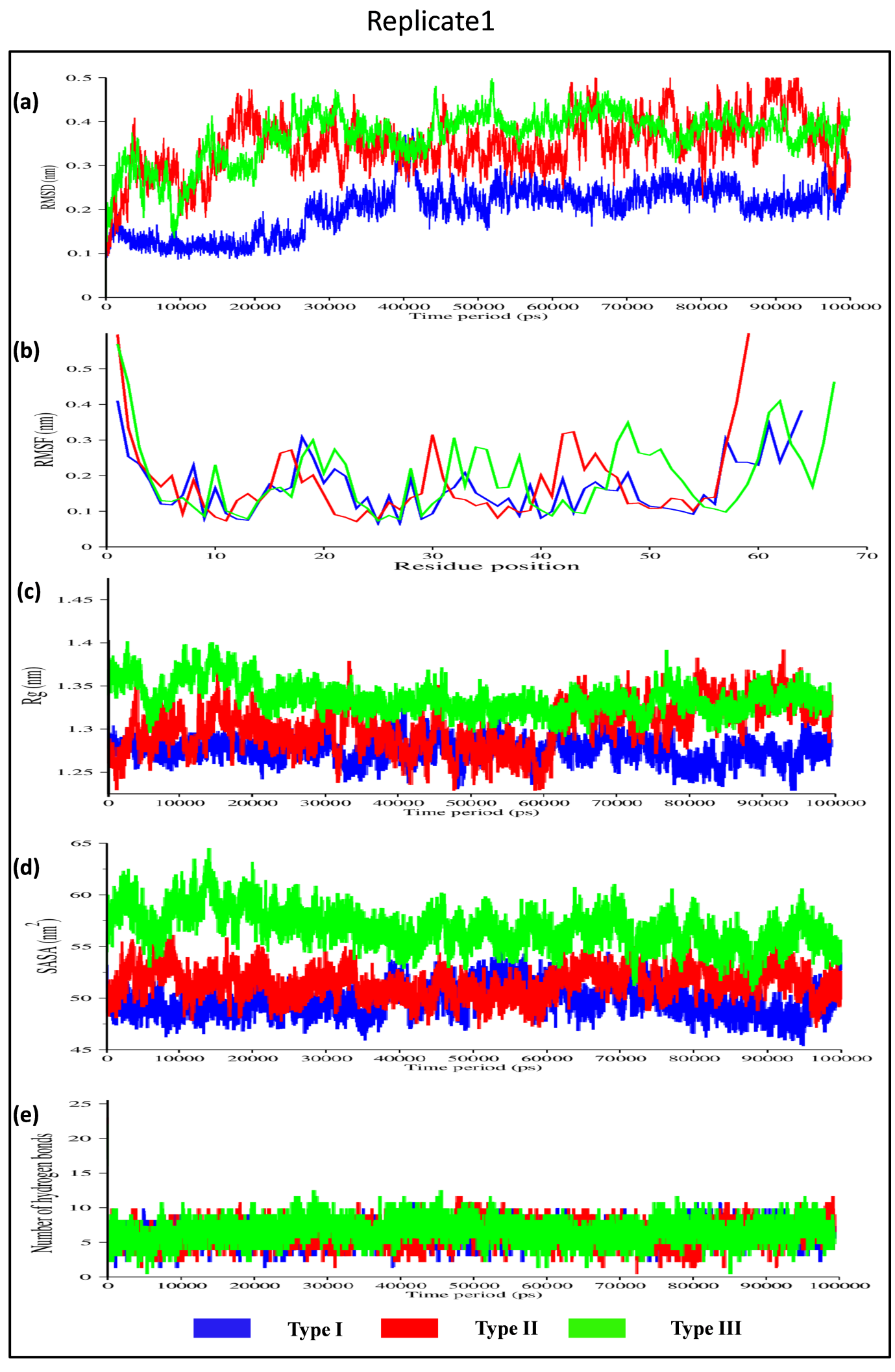


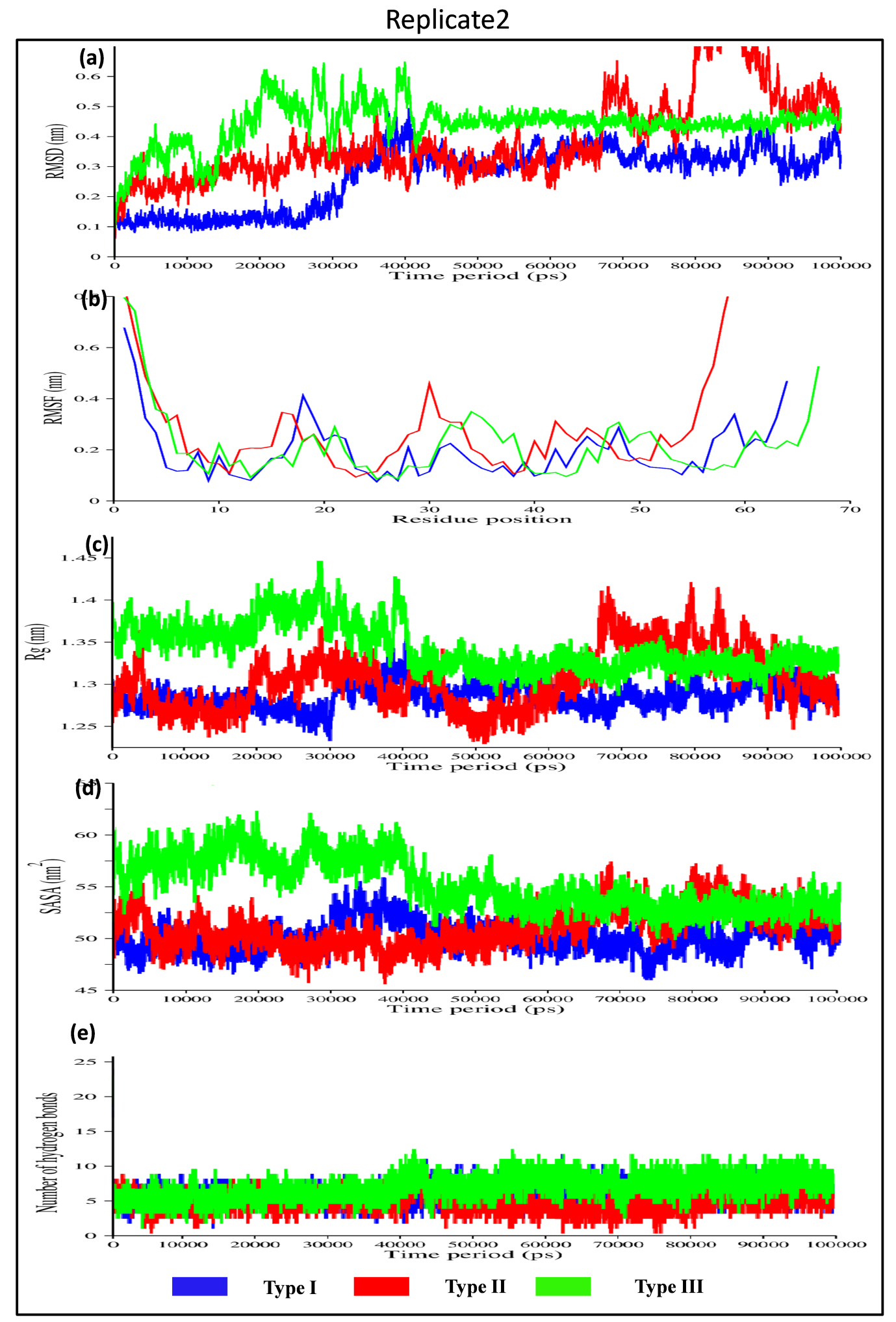

Supplement: Supplementary file 1 [file biology-08-00083-s001.zip › Supplementary Materials/Supple.figures/Figure S11.docx]
