# Supplementary material for: Understanding the Effect of Structural Diversity in WRKY Transcription Factors on DNA Binding Efficiency through Molecular Dynamics Simulation
Source: Biology (Basel). 2019 Nov 4;8(4):83. doi: 10.3390/biology8040083 (PMC6956055; doi:10.3390/biology8040083)
Supplement: Supplementary file 1 [file biology-08-00083-s001.zip › Supplementary Materials/Supple.tables/Table S3.docx]

**Table S3.** Superimposition of Type-I, Type-II and Type-III WRKY members and also with the templates to calculate the structural closeness in terms of global as well as local RMSD-values that showed the structural conservation of WRKY DBDs.

| **Superposition** | **Identity** | **Similarity** | **Local RMSD** | | **Global RMSD** | |
| --- | --- | --- | --- | --- | --- | --- |
|  |  |  | **α-carbons** | **Back bone** | **α-carbons** | **Back bone** |
| CcWRKY1&CcWRKY51 | 37/64 (57.8%) | 46/64 (71.9%) | 0.50 | 0.51 | 0.50 | 0.51 |
| CcWRKY1&CcWRKY70 | 22/67 (32.8%) | 33/67 (49.3%) | 1.54 | 1.48 | 1.54 | 1.48 |
| CcWRKY51&CcWRKY70 | 23/67 (34.3%) | 37/67 (55.2%) | 1.60 | 1.51 | 1.60 | 1.51 |
| CcWRKY1&2AYD | 51/76 (67.1%) | 61/76 (80.3%) | 0.35 | 0.46 | 0.35 | 0.46 |
| CcWRKY1&1WJ2 | 44/71 (62.0%) | 56/71 (78.9%) | 3.10 | 3.01 | 3.10 | 3.01 |
| CcWRKY51&2AYD | 35/76 (46.1%) | 47/76 (61.8%) | 0.47 | 0.52 | 0.47 | 0.52 |
| CcWRKY51&1WJ2 | 36/71 (50.7%) | 45/71 (63.4%) | 2.51 | 2.40 | 2.51 | 2.40 |
| CcWRKY70&2AYD | 23/79 (29.1%) | 34/79 (43.0%) | 1.55 | 1.46 | 1.55 | 1.46 |
| CcWRKY70&1WJ2 | 28/76 (36.8%) | 38/76 (50.0%) | 6.02 | 5.94 | 6.02 | 5.94 |
| 2AYD&1WJ2 | 46/76 (60.5%) | 61/76 (80.3%) | 8.86 | 8.75 | 8.86 | 8.75 |
