# Supplementary material for: Understanding the Effect of Structural Diversity in WRKY Transcription Factors on DNA Binding Efficiency through Molecular Dynamics Simulation
Source: Biology (Basel). 2019 Nov 4;8(4):83. doi: 10.3390/biology8040083 (PMC6956055; doi:10.3390/biology8040083)
Supplement: Supplementary file 1 [file biology-08-00083-s001.zip › Supplementary Materials/Supple.tables/Table S2.docx]

**Table S2.** Comparative secondary structure analysis of target and templates predicted from Self-Optimized Prediction Method with Alignment (SOPMA) Server

| **SOPMA** | **CcWRKY1** | **CcWRKY51** | **CcWRKY70** | **2AYB** | **1WJ2** |
| --- | --- | --- | --- | --- | --- |
| Alpha helix (Hh) | 18.75% | 16.67% | 13.43% | 13.16% | 17.95% |
| 3_10_  helix (Gg) | 0.00% | 0.00% | 0.00% | 0.00% | 0.00% |
| Pi helix (Ii) | 0.00% | 0.00% | 0.00% | 0.00% | 0.00% |
| Beta bridge (Bb) | 0.00% | 0.00% | 0.00% | 0.00% | 0.00% |
| Extended strand (Ee) | 23.44% | 23.33% | 25.37% | 28.95% | 19.23% |
| Beta turn (Tt) | 12.50% | 10.00% | 4.48% | 13.16% | 12.82% |
| Bend region (Ss) | 0.00 | 0.00% | 0.00% | 0.00% | 0.00% |
| Random coil (Cc) | 45.31% | 50.00% | 56.72% | 44.74% | 50.00% |
| Ambiguous states (?) | 0.00 | 0.00% | 0.00% | 0.00% | 0.00% |
| Other states | 0.00 | 0.00% | 0.00% | 0.00% | 0.00% |
