# Supplementary material for: Understanding the Effect of Structural Diversity in WRKY Transcription Factors on DNA Binding Efficiency through Molecular Dynamics Simulation
Source: Biology (Basel). 2019 Nov 4;8(4):83. doi: 10.3390/biology8040083 (PMC6956055; doi:10.3390/biology8040083)
Supplement: Supplementary file 1 [file biology-08-00083-s001.zip › Supplementary Materials/Supple.tables/Table S1.docx]

| **Type** | **Accession no.** | **Length** | **M. wt.(kDa)** | **pI** | **II** | **EC** | **AI** | **GRAVY** |
| --- | --- | --- | --- | --- | --- | --- | --- | --- |
| Type-I | XP_020234360.1 | 60 | 7017.84 | 9.47 | 17.71 | 13075 | 51.83 | -1.252 |
| Type-II | XP_020213367.1 | 60 | 7079.85 | 9.05 | 28.31 | 14565 | 30.67 | -1.523 |
| Type-III | XP_020210665.1 | 63 | 7528.51 | 8.75 | 39.92 | 13200 | 49.52 | -0.933 |

**Table S1.** Physicochemical characteristics of selected accessions of Type I, Type II, and III WRKYs from pigeonpea.
