# Supplementary figures and images for: Understanding the Effect of Structural Diversity in WRKY Transcription Factors on DNA Binding Efficiency through Molecular Dynamics Simulation
Source: Biology (Basel). 2019 Nov 4;8(4):83. doi: 10.3390/biology8040083 (PMC6956055; doi:10.3390/biology8040083)

Qualitative Model Energy Analysis (QMEAN) measurements

Type I


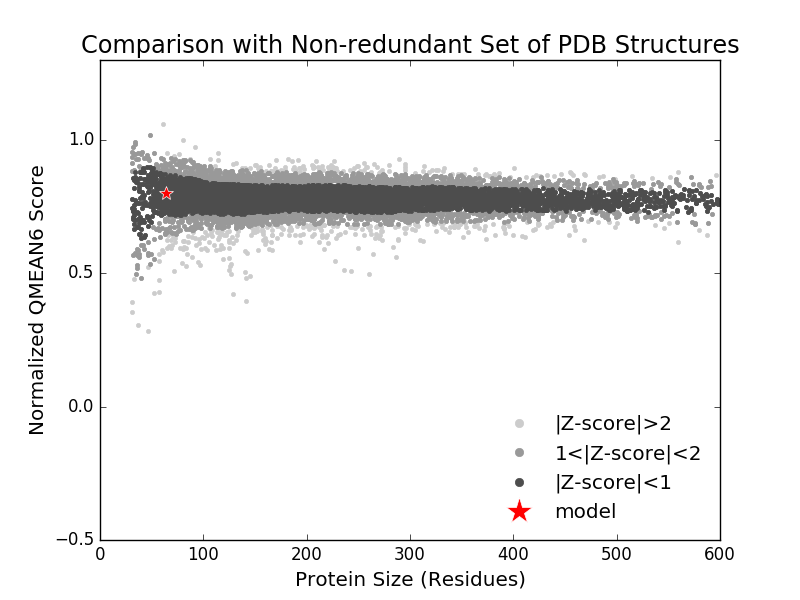


Type II


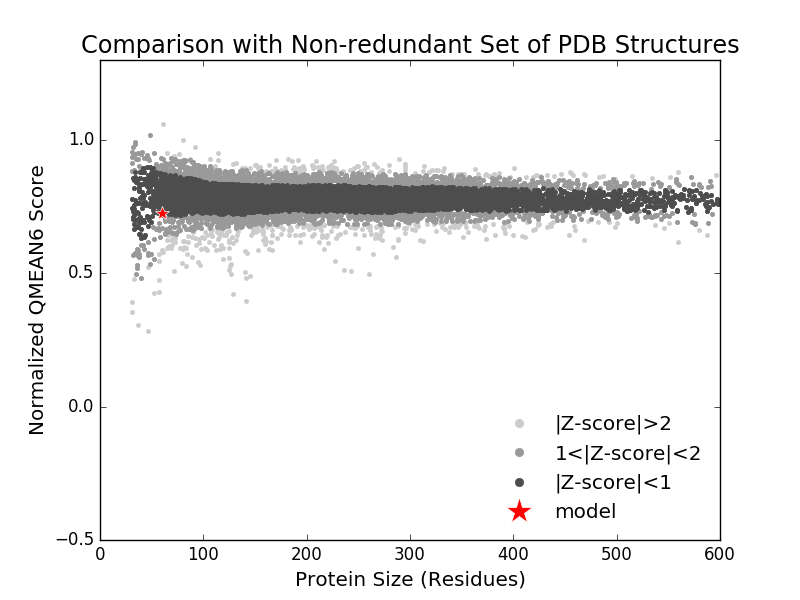


Type III


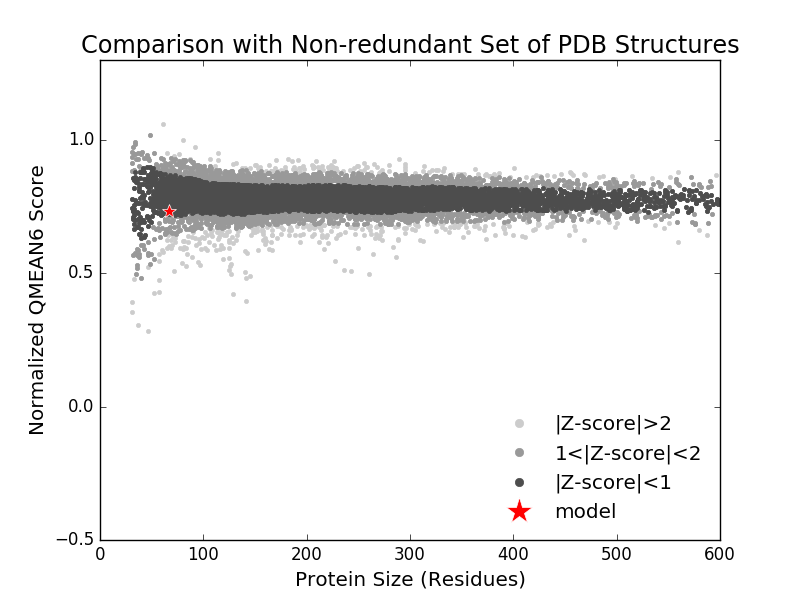

Supplement: Supplementary file 1 [file biology-08-00083-s001.zip › Supplementary Materials/Supple.figures/Figure S9.docx]

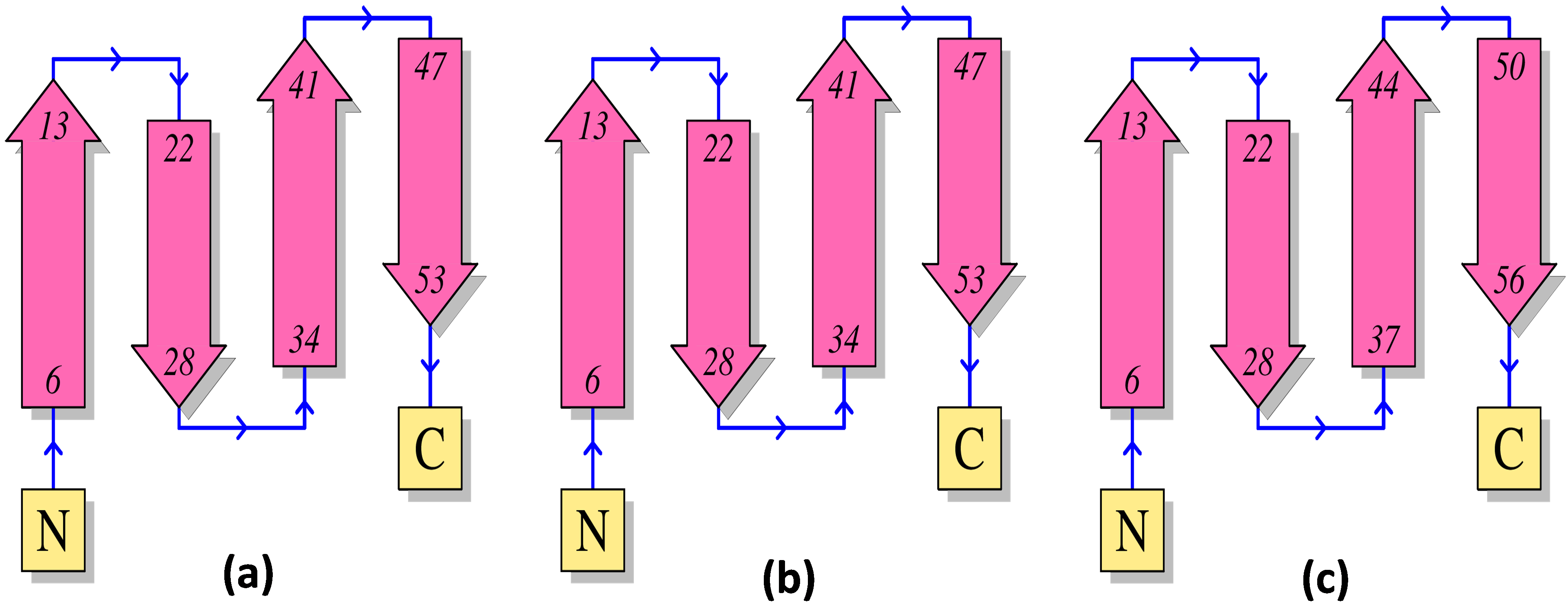

Supplement: Supplementary file 1 [file biology-08-00083-s001.zip › Supplementary Materials/Supple.figures/Figure S5.bmp]

Varify Results

Type I


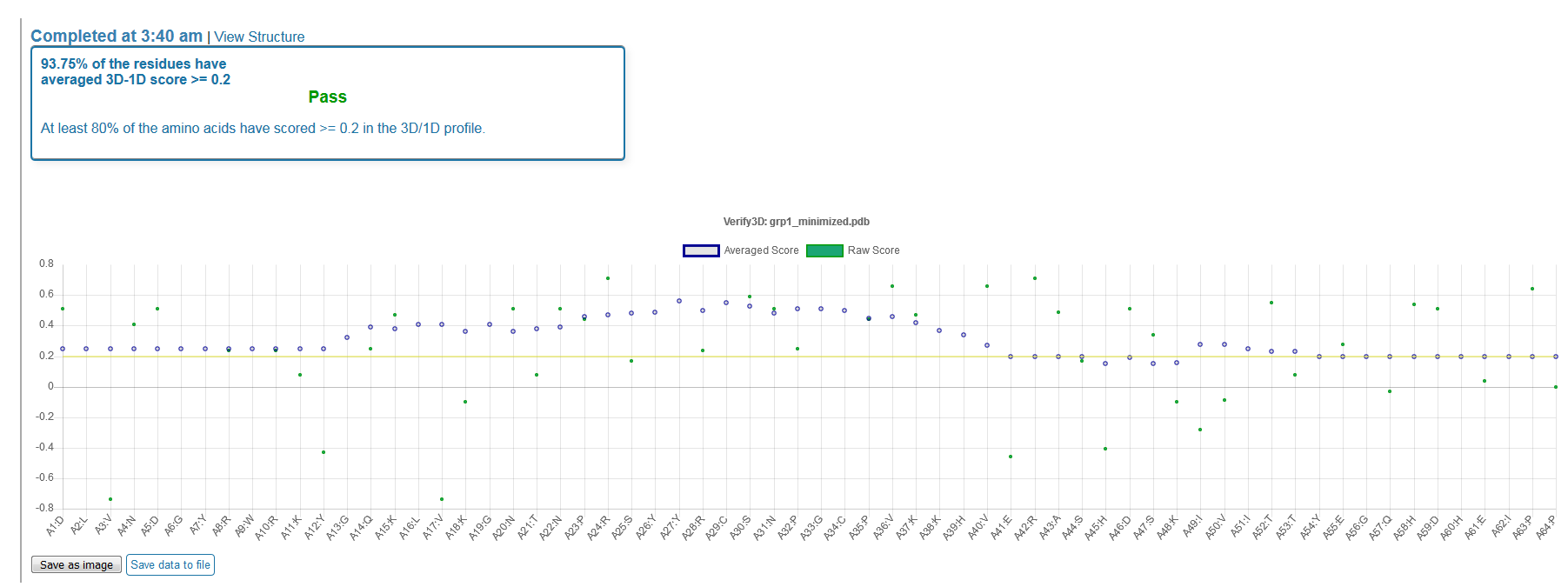


Type II


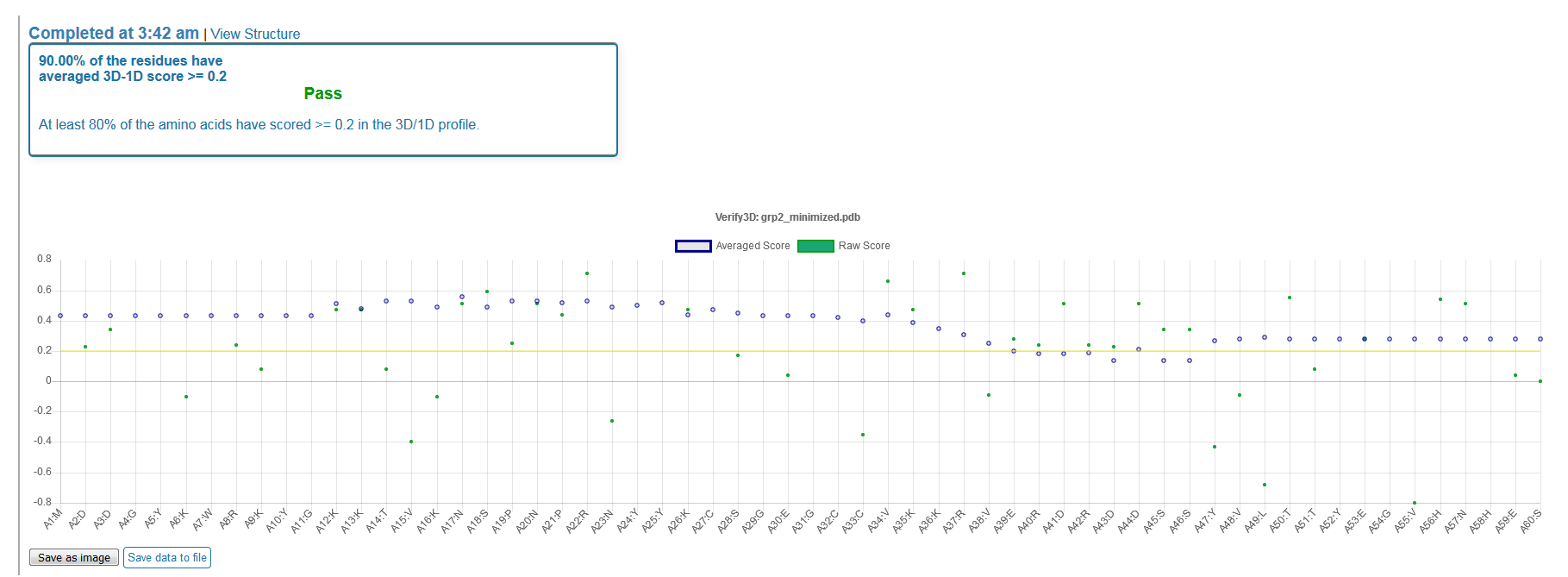


Type III


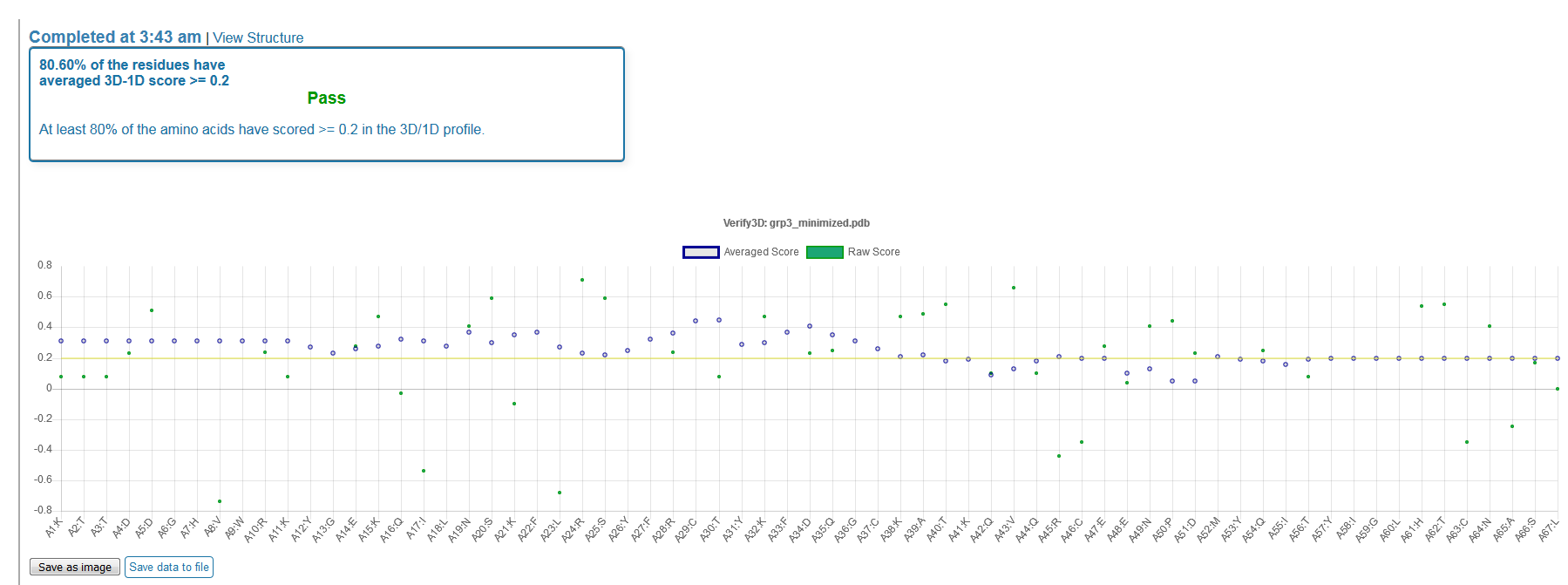

Supplement: Supplementary file 1 [file biology-08-00083-s001.zip › Supplementary Materials/Supple.figures/Figure S8.docx]

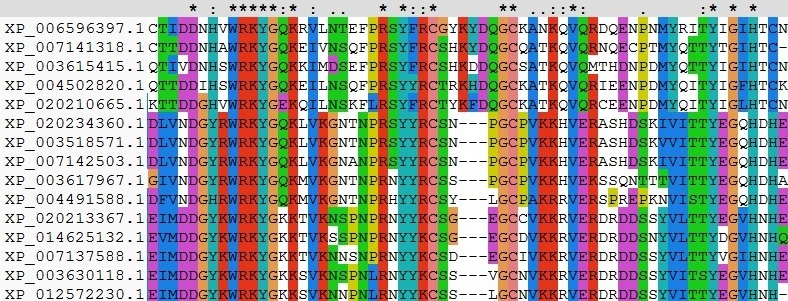

Supplement: Supplementary file 1 [file biology-08-00083-s001.zip › Supplementary Materials/Supple.figures/Figure S4.tif]

ERRAT Score graph

Type I


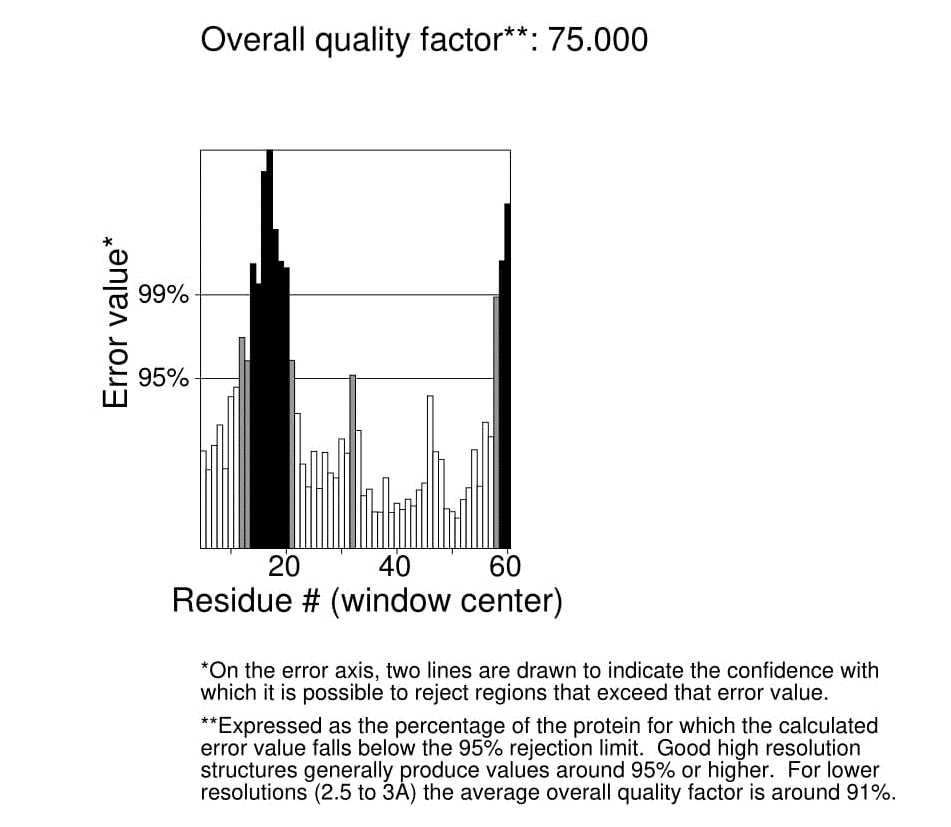


Type II


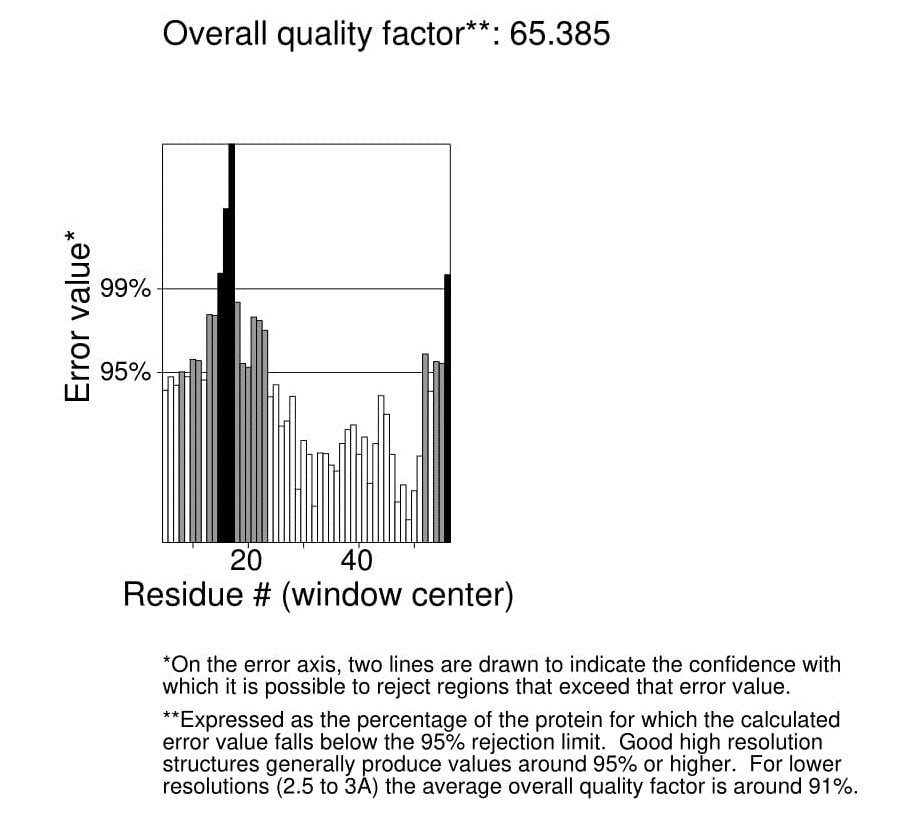


Type III


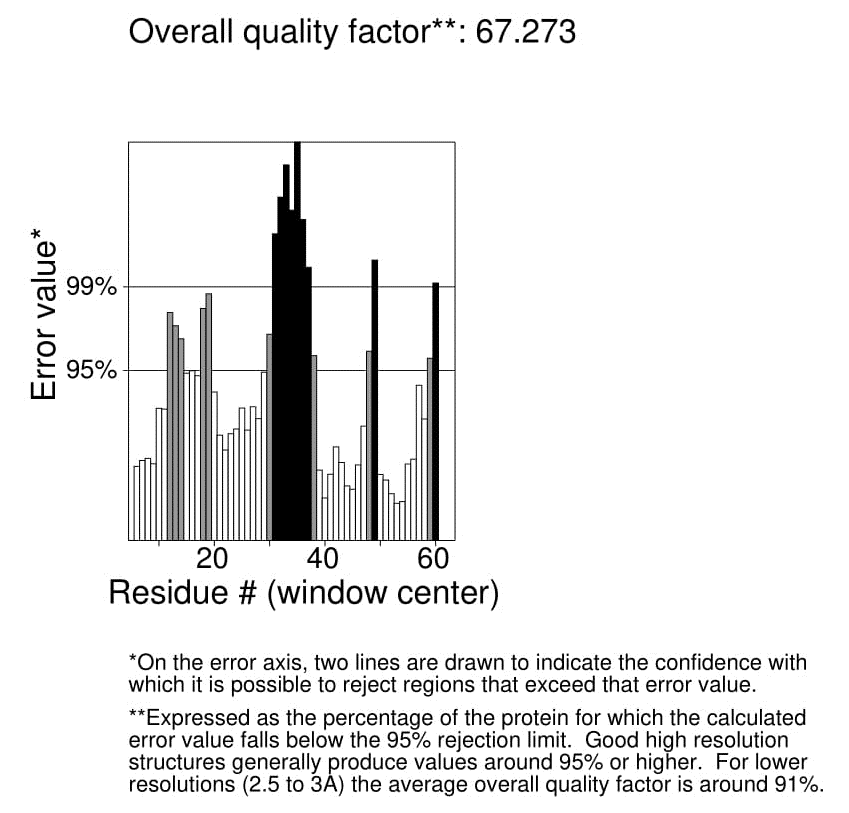

Supplement: Supplementary file 1 [file biology-08-00083-s001.zip › Supplementary Materials/Supple.figures/Figure S7.docx]

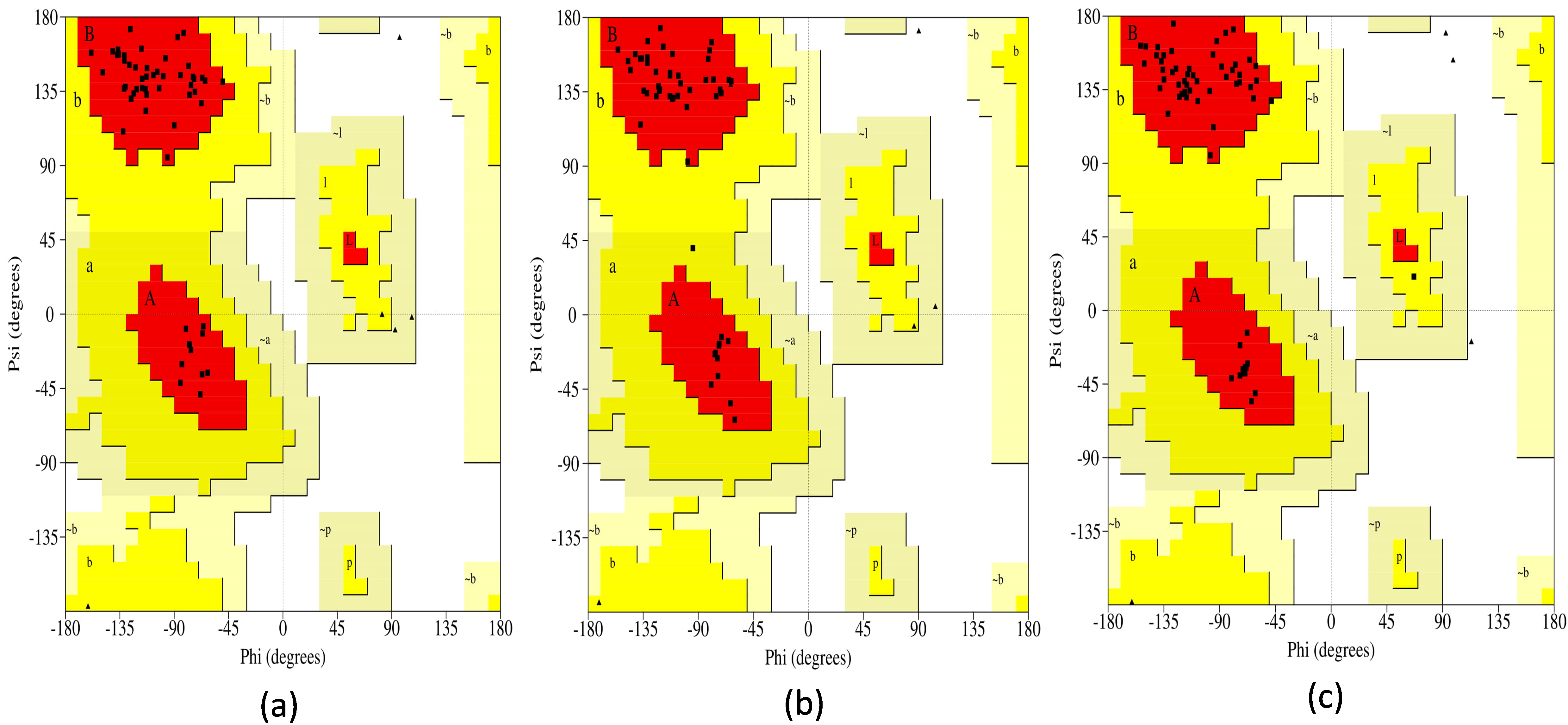

Supplement: Supplementary file 1 [file biology-08-00083-s001.zip › Supplementary Materials/Supple.figures/Figure S6.tif]

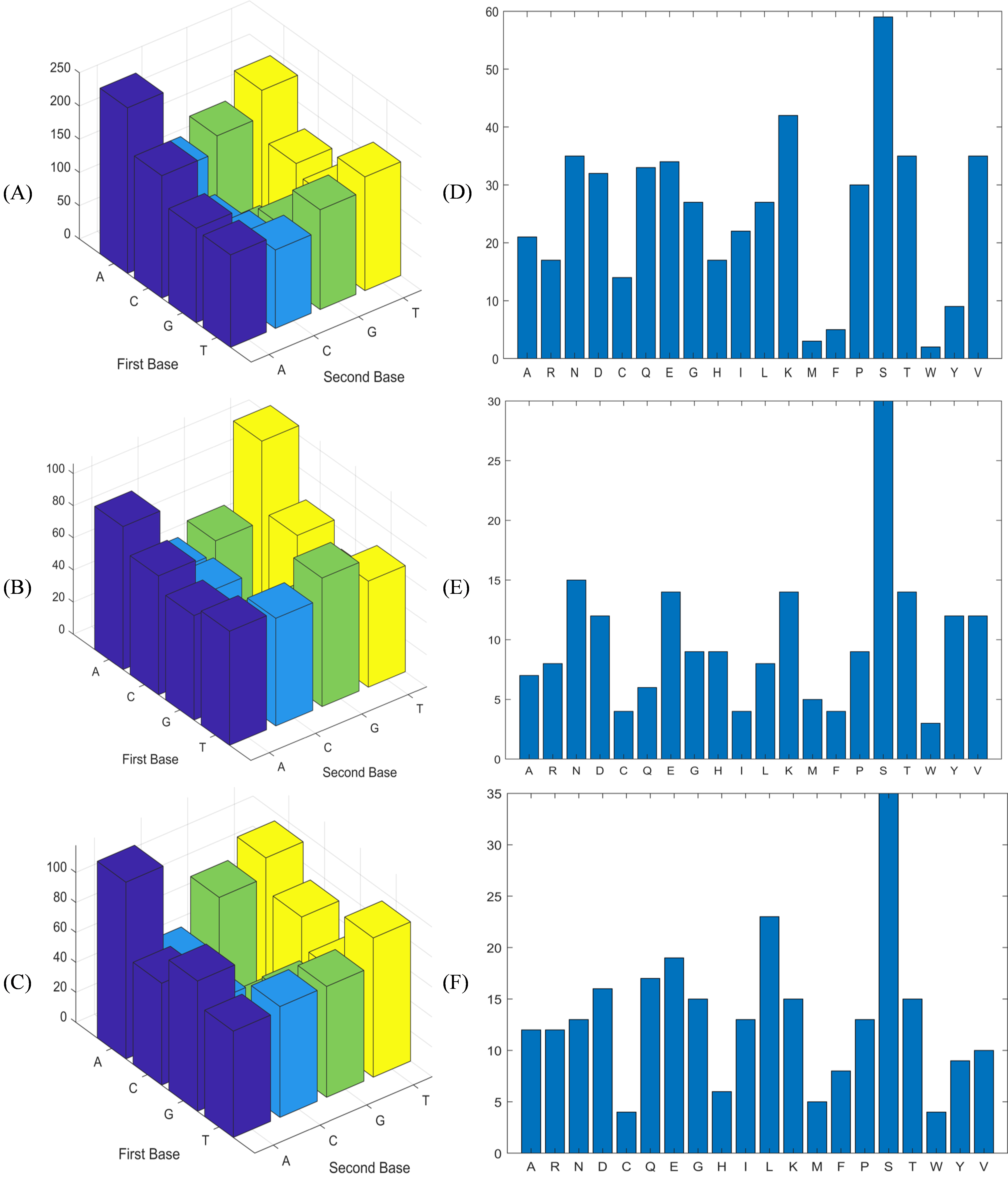

Supplement: Supplementary file 1 [file biology-08-00083-s001.zip › Supplementary Materials/Supple.figures/Figure S2.tif]

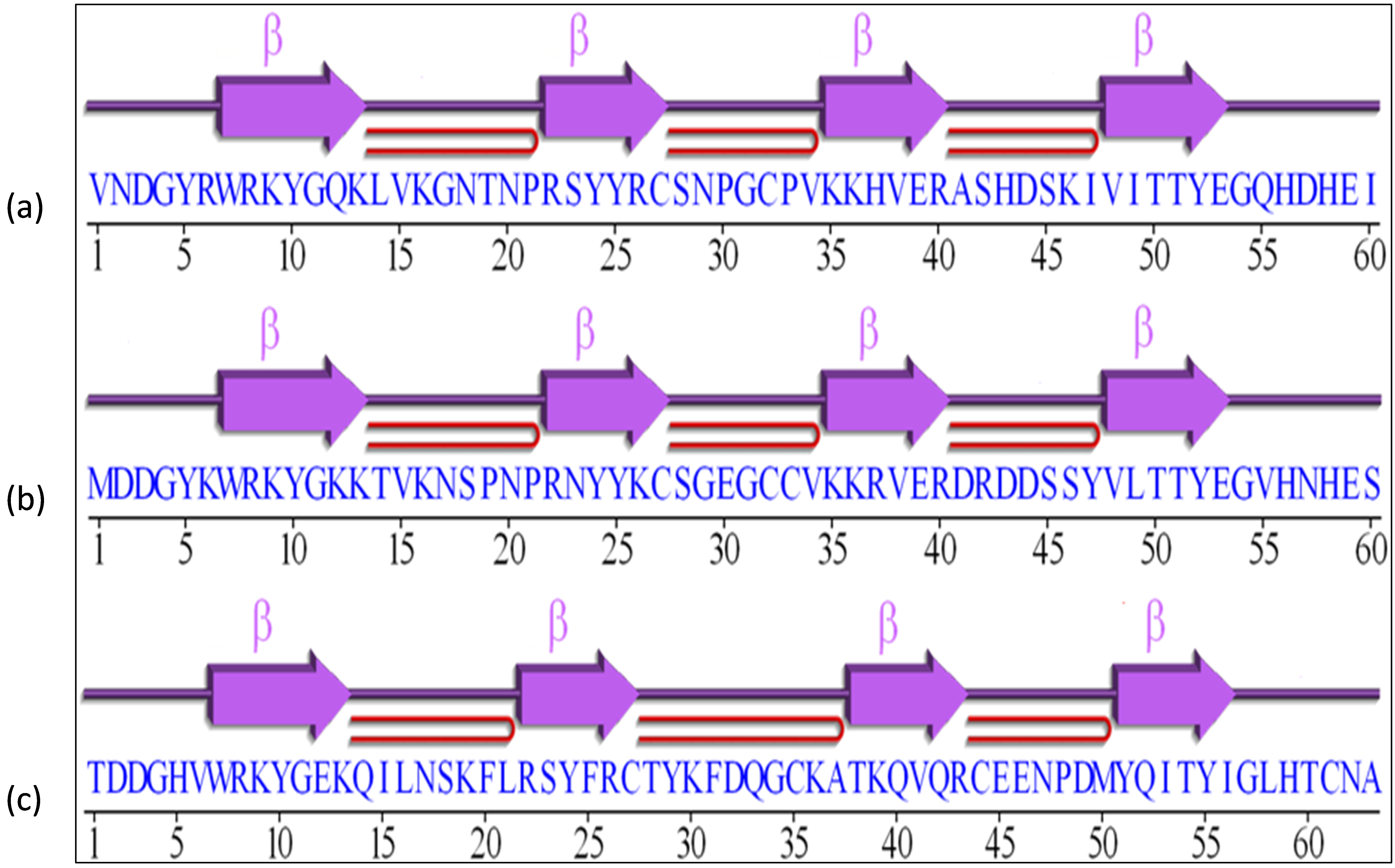

Supplement: Supplementary file 1 [file biology-08-00083-s001.zip › Supplementary Materials/Supple.figures/Figure S3.tif]

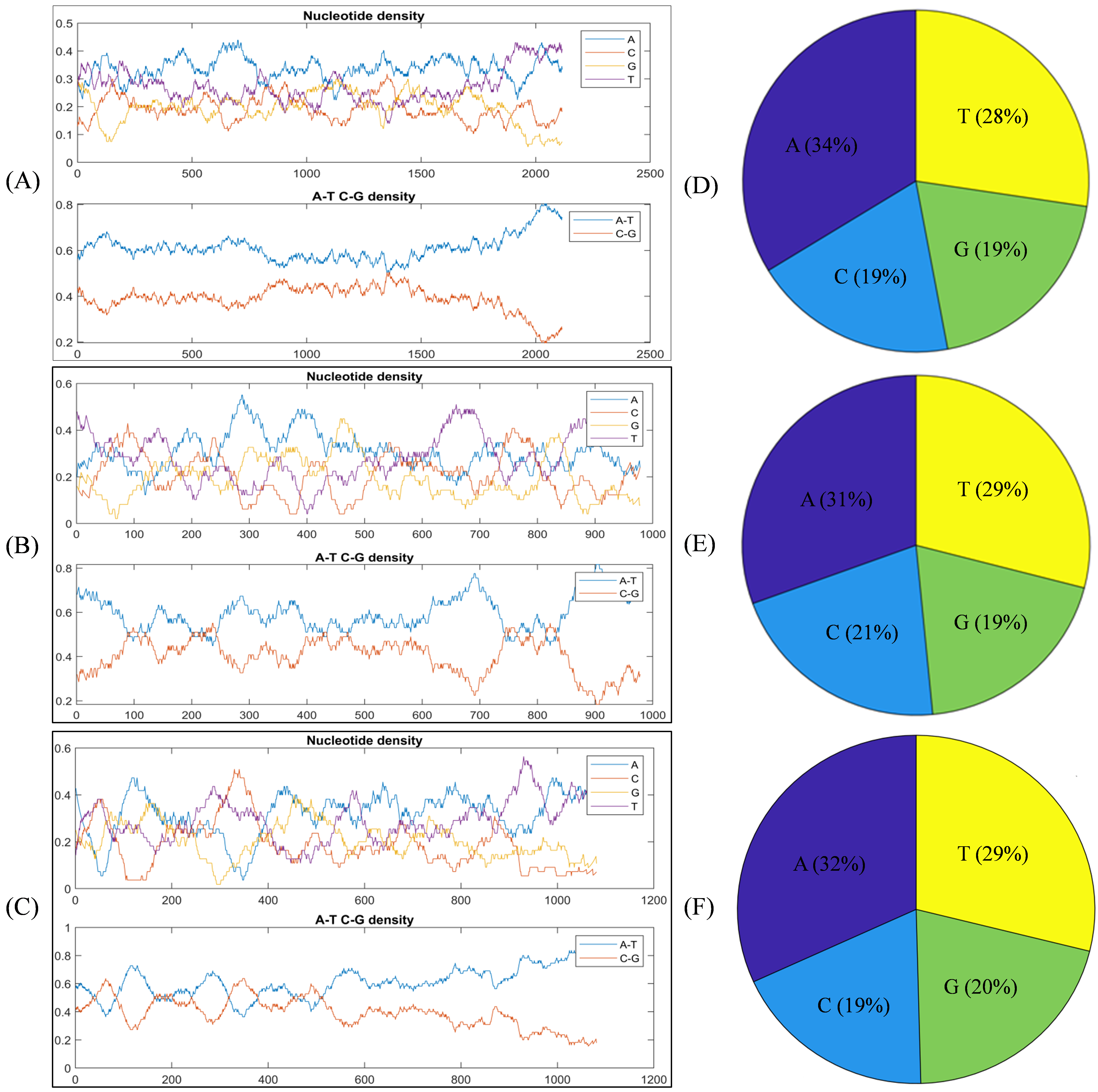

Supplement: Supplementary file 1 [file biology-08-00083-s001.zip › Supplementary Materials/Supple.figures/Figure S1.tif]

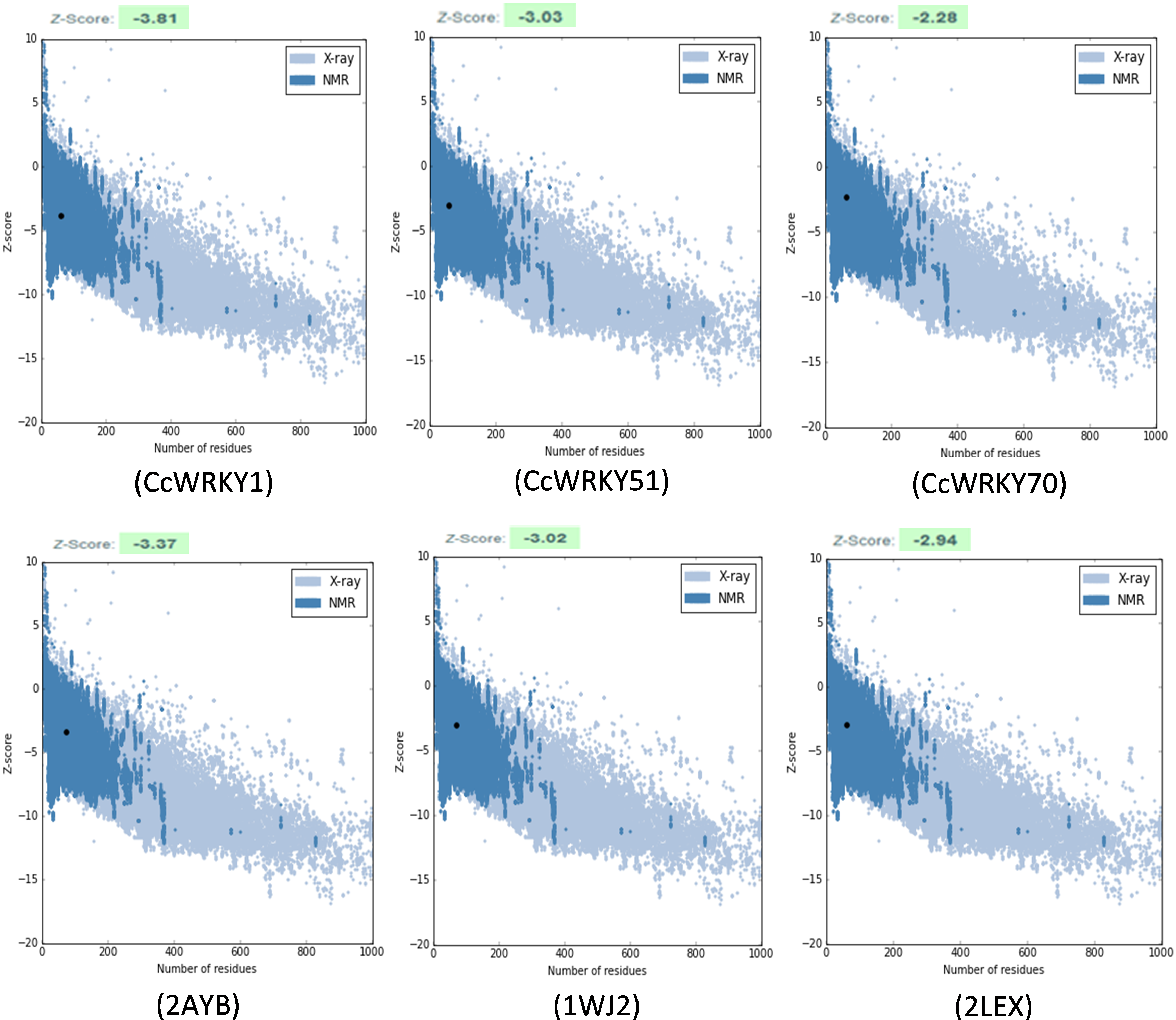

Supplement: Supplementary file 1 [file biology-08-00083-s001.zip › Supplementary Materials/Supple.figures/Figure S10.tif]
